# Systematic Determination of Replication Activity Type Highlights Interconnections between Replication, Chromatin Structure and Nuclear Localization

**DOI:** 10.1371/journal.pone.0048986

**Published:** 2012-11-07

**Authors:** Shlomit Farkash-Amar, Yaara David, Andreas Polten, Hadas Hezroni, Yonina C. Eldar, Eran Meshorer, Zohar Yakhini, Itamar Simon

**Affiliations:** 1 Department of Microbiology and Molecular Genetics, Hebrew University Medical School, IMRIC, Jerusalem, Israel; 2 Department of Electrical Engineering, Technion – Israel Institute of Technology, Haifa, Israel; 3 Agilent Technologies, Waldbronn, Germany; 4 Department of Genetics, Hebrew University, Jerusalem, Israel; 5 Department of Computer Sciences, Technion – Israel Institute of Technology, Haifa, Israel; 6 Agilent Laboratories Tel-Aviv, Israel; The University of Nottingham, United Kingdom

## Abstract

DNA replication is a highly regulated process, with each genomic locus replicating at a distinct time of replication (ToR). Advances in ToR measurement technology enabled several genome-wide profiling studies that revealed tight associations between ToR and general genomic features and a remarkable ToR conservation in mammals. Genome wide studies further showed that at the hundreds kb-to-megabase scale the genome can be divided into constant ToR regions (CTRs) in which the replication process propagates at a faster pace due to the activation of multiple origins and temporal transition regions (TTRs) in which the replication process propagates at a slower pace. We developed a computational tool that assigns a ToR to every measured locus and determines its replication activity type (CTR versus TTR). Our algorithm, ARTO (Analysis of Replication Timing and Organization), uses signal processing methods to fit a constant piece-wise linear curve to the measured raw data. We tested our algorithm and provide performance and usability results. A Matlab implementation of ARTO is available at http://bioinfo.cs.technion.ac.il/people/zohar/ARTO/. Applying our algorithm to ToR data measured in multiple mouse and human samples allowed precise genome-wide ToR determination and replication activity type characterization. Analysis of the results highlighted the plasticity of the replication program. For example, we observed significant ToR differences in 10–25% of the genome when comparing different tissue types. Our analyses also provide evidence for activity type differences in up to 30% of the probes. Integration of the ToR data with multiple aspects of chromosome organization characteristics suggests that ToR plays a role in shaping the regional chromatin structure. Namely, repressive chromatin marks, are associated with late ToR both in TTRs and CTRs. Finally, characterization of the differences between TTRs and CTRs, with matching ToR, revealed that TTRs are associated with compact chromatin and are located significantly closer to the nuclear envelope. Supplementary material is available. Raw and processed data were deposited in Geo (GSE17236).

## Introduction

Replication of the DNA occurs in the S phase of the cell cycle in a controlled and organized manner. The controlled nature of the replication order was originally established based on measuring the time of replication (ToR) of many individual loci. In recent years, genome-wide measurement approaches have greatly improved our understanding of this controlled process (reviewed in [1]). Genome-wide ToR profiling studies enables an analysis of the global properties of ToR. The ToR of a genomic region is usually invariable in the same tissue and is highly conserved between mammals [2], [3]. However, ToR shows considerable amount of plasticity between tissue types [2], [4], [5], [6], [7]. Analysis of the association between the ToR and general genomic features revealed that early replication is associated with high GC content, high gene density, transcription potential and open chromatin marks whereas late replication is associated with the opposite features (reviewed in [1]). Existing analyses of ToR association to other properties suggest that the ToR reflects high order organization of the chromosomes but they fall short in addressing any mechanistic questions regarding the relationships between the ToR and the other traits.

DNA replication is organized into two basic replication activity types. The basic units of replication are replicons, defined as the region copied by the activation of a single origin of replication. Adjacent origins are usually activated in a coordinated manner and thus large genomic regions replicate at approximately the same time [4], [8], [9]. This replication activity type is hereby termed Constant ToR Regions (CTRs) to reflect the fact that within them the ToR is quite constant. Regions that manifest another type of replication activity type, namely a gradual change of ToR are called Temporal Transition Regions (TTRs [10]). One such region was identified more than a decade ago in the mouse IgH locus of non-B cells, in which it serves as a transition region between early and late CTRs [11], [12]. Recent studies that used genome-wide measurement approaches found that TTRs are a common replication activity type and that they occur in almost all cases of temporal transition between CTRs [1], [4], [6], [7], [9]. It is unclear if the temporal transitions in TTRs are the result of sequential activation of a series of origins [13] or of a single long replication fork (reviewed in [1]). For the purpose of our study it is important to note that under both models it is true that actual ToR within TTRs is determined and controlled by the distance from an independently activated origin.

The importance of precisely mapping TTRs at multiple tissues was highlighted by a recent study that demonstrated the FRA3B fragile site to be located within a tissue specific TTR. Furthermore, FRA3B fragility has shown dependency on the replication activity type of the region [14]. Nevertheless, no systematic effort was so far taken to characterize TTRs in multiple tissue types, partly due to the lack of computational tools for distinguishing between CTRs and TTRs. In this work we combined precise mapping of the ToR in multiple mouse and human tissues, using genome-wide measurements, with the development of a computational tool that for every measured region determines its ToR and its replication activity type (CTR versus TTR). Methods for determining ToR from raw measurement data were presented in the past but little work is reported which addresses computational methods for the determination of CTRs and TTRs. We make model assumptions that enable this classification into CTRs and TTRs and thereby the further analysis that is performed in our study. Previous work by Guilbaud et al [13] used sequencing reads form BrdU labeled DNA from four S phase compartments to estimate S_50_, the fraction of the S phase duration at which 0.5 of all DNA at a defined region has been replicated. The resulting ToR signal is an interpolated version of the pointwise measurement values. The authors continue and determine CTRs and TTRs by considering the inferred apparent speed of replication. This computational approach to determining CTRs and TTRs, taken by Guilbaud et al, therefore makes different model assumptions than the one we present herein.

Applying our model driven algorithm to the ToR data, we observed considerable plasticity, across different tissue types, in the genomic organization of the replication program. We also analyzed the association between the ToR and various genomic features both in TTRs and CTRs. Our analysis suggests that the ToR has a role in shaping chromatin structure and that the replication activity type is evidently influenced by nuclear localization, such as lamina proximity.

## Results

### Measuring ToR in Multiple Mouse and Human Tissue Types

We have previously measured the ToR of the entire human genome in primary foreskin fibroblasts (FFT) and in Molt4 lymphoblastic cell line and of the entire mouse genome in mouse embryonic fibroblasts (MEFs) and in L1210 lymphoblastic cell line [3], [9]. Here we expanded the analysis to two additional primary mouse tissues, namely mouse ES (Embryonic Stem) cells and NPCs (Neuronal Progenitor Cells) derived from them. Our results are consistent with previous knowledge about ToR including its regional nature (all samples showed high auto correlation, see examples in Figure S1) and a high correlation with the regional GC content, gene density and transcription (Figure S2). Similar to previous reports [4], in ES cells the correlation of ToR with both gene density and GC content was much lower (R = 0.18 and R = 0.36, respectively) than in the other cell types (R>0.38 and R>0.6, respectively).

### Data Analysis

**Figure 1:**
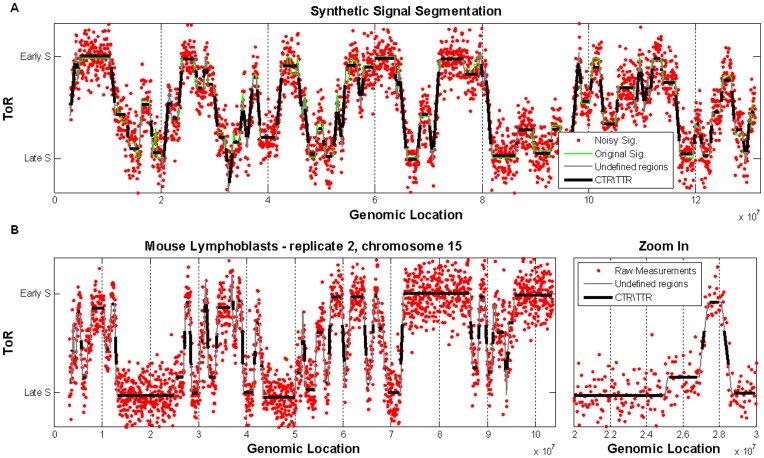
Generation of ToR maps with the ARTO algorithm. (A) Noise was added to synthetic signals (original signals - green; noisy signals – red) and the data was segmented using ARTO (black and grey lines). (B) Raw ToR measurements of chromosome 15 in mouse Lymphoblasts replicate 2 (red), and its segmentation using ARTO (black and gray). On the right – zoom in on 1 Mb region. The undefined regions are regions close to transitions between CTRs and TTRs (see Methods). The Y axis represents normalized S/G1 ratios as described in the Methods section and in [3]. S/G1 ratios range from 1 to 2.

As has been described before [4], [6], [7], [9], [13], the replication program is organized in two types of regions – CTRs (Constant ToR Regions) and TTRs (Temporal Transition Regions). These two replication activity types are represented by two different types of segments in the ToR maps (Figure 1). While the CTRs are represented by horizontal lines, the TTRs are represented by diagonal lines with different slopes that correspond to the rate of replication propagation. A more general distinction would be to consider fast propagation of replication versus slow propagation of replication. For simplicity of presentation and for reasons related to the computational complexity we chose to represent fast propagation by constant ToR. We further discuss this issue in the discussion section. To automatically analyze ToR data and assign to each genomic locus both its ToR and its replication activity type, we developed a novel algorithm dedicated to the Analysis of Replication Timing and Organization (ARTO). This algorithm is based on the following model assumptions –.

- Genomic ToR maps are composed of regions representing the two propagation rates – fast propagation and slow propagation. For computational simplicity we further assume that they are piecewise linear;
- There are no replication fork barriers (based on our observation that these are very rare [1]). The ToR maps are therefore continuous.
- The slopes in the slow propagating regions should be within a certain range of values (0.25–4 Kb/minute [9]). The parameter that controls the slope can be adjusted to accommodate different model assumptions, including the effects described in [13] (see Supplementary Methods and Figure S3).

We chose to align ourselves with common nomenclature and call the two activity types CTRs and TTRs.

Based on these model assumptions our approach for reconstructing the ToR signal out of its noisy per-probe array-based raw measurements is as follows. *First*, we utilized pre-processing steps that include noise filtering and parameter setting. *Next*, we divided the signal into overlapping windows with a constant number of data points. For each window we used the Hough transform [15], [16] to search for potential lines that match some of the points in the window and are either constant or have a slope within the given fork-rate range. Based on these lines we computed an approximate best-fit piecewise-linear continuous segmentation for the window using dynamic programming. *Finally*, each window was set to start at the last breakpoint of the previous window. We continued to compute the ToR at each window, until reaching the last measurement point. Applying ARTO to raw genomic ToR data provided a continuous piecewise linear ToR map of the entire measured region of the genome, which represents the best fit to the raw measurement data. Furthermore, we utilized ARTO to classify each genomic region into either TTR or CTR. An alternative approach to determining CTRs and TTRs is described in Guilbaud et al [13].

We evaluated the performance of ARTO using simulated data and found that even when we introduced 15% noise (which corresponds to noise levels estimated in actual data, see Methods), ARTO can recover accurate ToR for the entire (simulated) genome (mean RMSE of 100 synthetic simulated signals, each 3200 probes long, is 5%; see Supplementary Methods for precise definitions). The classification into CTRs and TTRs was also fairly accurate, depending on the distance from the closest inferred breakpoint. The fraction of probes (in simulated data) that were correctly classified at a distance >120 Kb in CTRs and >180 Kb in TTRs ranges from 80% to over 95% of probes (Figure 1a; Methods). By incorporating this information into ARTO we can now analyze raw data and compute, for each genomic locus, its ToR and an assignment to one of three replication activity type options - CTR, TTR or undefined (Figure 1b; Methods). Additional evidence for the good quality of the performance of ARTO stems from considering the ToR assigned to each genomic locus in biological replicates. The correlations between replicates increased significantly after genome segmentation by ARTO (Figure S4). A recent study [17] recommend the use of auto correlations as one approach to assessing ToR data quality. Indeed auto correlation analysis confirms the quality of our ToR measurements and the accuracy of the ARTO algorithmic approach (Figure S1).

For a detailed description of ARTO, including assumptions, performance results and a full description of all steps see Supplementary Methods. We also report testing the effect of selecting different algorithm parameters in Supplementary Methods. A Matlab implementation of ARTO is available at http://bioinfo.cs.technion.ac.il/people/zohar/ARTO/. ARTO results for the four mouse and the two human cell lines can be found in Tables S1 and S2 respectively.

### Comparison between Tissue Types

Measuring the ToR at multiple tissue types and multiple organisms using exactly the same measurement methodology and protocols allows us to address questions regarding replication organization plasticity both at the level of the ToR and at the level of the replication activity type.

**Figure 2:**
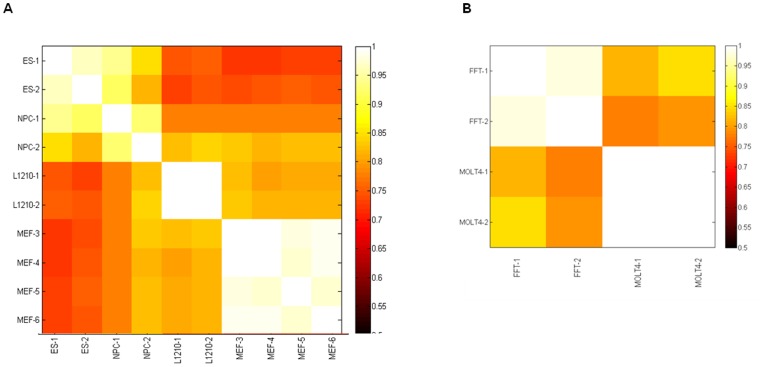
Comparison of the ToR between tissues. The fraction of probes with similar ToR is shown for each pair of mouse tissues in A and human tissues in B. Probes were defined as having similar ToR as long as the ToR difference between the 2 tissues was less than 30% of the cycles phase length. As expected, replicates of the same tissue show higher percentage of similarity (90–100%) while pairs of different tissues present lower similarity level (70–80%).

We found a significant ToR inter-tissue plasticity. A considerable change in the ToR (>30% of the S phase length) occurs quite frequently (10–25% of the genome) between tissue types while it is quite rare (1.5–8%) within replicates of the same tissue type (Figure 2). We noted, however, that one of the NPC duplicates (NPC1) was very similar to the ES samples and somewhat different from the other NPC sample (Figure 2 and Figure S3), possibly indicating a poor differentiation stage of this sample. This sample was excluded from further analyses.

Compilation of the data of the four mouse tissue types revealed that in most of the genome (56%) ToR changes when different tissue types are compared. This percentage is similar to that found by previous studies who compared the ToR of four and ten different tissue types and found that approximately 50% of the genome changes its ToR [6].

Applying ARTO to the L1210 dataset results in 13% of the genome annotated as TTRs. This estimation is similar to our previous estimation of 10% [9] where the measurement protocols and the analysis approaches used were different than the current ones. Expanding this analysis to additional human and mouse tissues revealed that in different tissue types a different proportion of the genome is replicated as a TTR (Human fibroblasts - 24%; mouse fibroblasts –37%; human lymphoblasts –33%; mouse ES cells –11%; mouse NPCs –19%).

It has been reported that upon differentiation of B cells a dormant origin within the immunoglobulin heavy chain TTR region is activated [10]. Importantly, our analysis allows the generalization of this observation. A direct comparison of the replication activity type in several mouse and human tissues revealed a substantial plasticity in the replication activity type. We found that in any pair of tissue types, 10–30% of the probes change their status from TTRs to CTRs, whereas the rate of status change between replicates of the same tissue type ranges from 0.5% to 10% (Table S3 and Figure S5), suggesting that the differential activation of origins is a common mechanism, in the context of development and differentiation processes.

Our analyses reveal that both the ToR and the replication activity type show a considerable amount of plasticity. In order to check whether these two features of the replication program are associated with each other, as expected, we analyzed the changes in the ToR between fibroblasts and lymphoblasts (both in human and in mouse) in light of the changes in the replication activity type. We found that regions that changed their ToR are highly enriched for regions that also changed their replication activity type both in human and in mouse (50% of all the regions that change their ToR also change their replication activity type while only 26% out of all regions change their replication activity type in human; similar results in mouse 56% versus 21%; for both cases the hyper geometric p value is <10^−300^). This association between replication activity type plasticity and ToR plasticity is very interesting and may shed some light on the basic building blocks of the replication maps (see Discussion).

### ToR and Other Genomic Properties

Measuring the ToR at multiple tissue types allows us to integrate it with other genomic features such as chromatin structure, DNA methylation, lamin association and DNase-I hyper sensitive sites, each measured in one or several tissue types that overlap our sample set.

It has been previously demonstrated that the ToR is associated with several basic properties of any given genomic locus, including its accessibility (DNase-I, [6]), its nuclear localization (lamin, [6]) and its participation in long range interactions (HiC, [2]). We have repeated these analyses with our human ToR data and obtained similar results (DNase-I, R = 0.53; HiC R = 0.66; Lamin R = −0.28; positive correlations mean that early replicating regions tend to have higher signals).

To analyze the association between ToR and DNA methylation we used the recently published methylome data [18]. To this end we used our human fibroblast ToR data and compared it to the methylome data (measured in a similar cell type). We have found that ToR and global methylation in fibroblasts are significantly correlated (R = 0.39), suggesting that early replicating regions are more methylated than late replicating loci. This correlation is not a simple consequence of the higher methylation levels observed in the body of genes [19], since we found higher correlation in intergenic regions (>20 Kb from genes; R = 0.32) than in genes (R = 0.21). Our results confirm previous studies [20], [21] and suggest a link between DNA methylation and ToR which is independent of gene body methylation [19].

**Figure 3:**
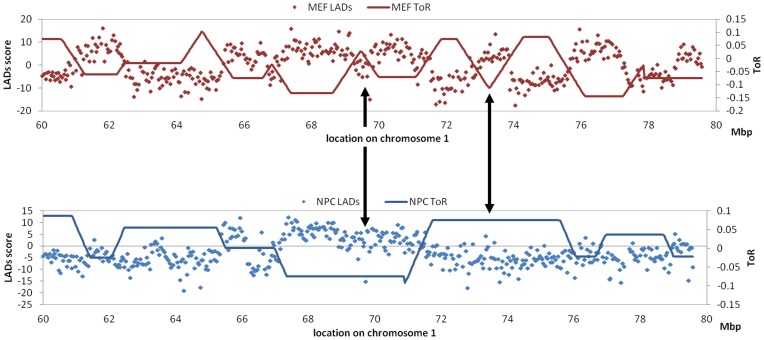
Correspondence between changes in ToR and changes in Lamina-Associated Domains (LADs). A region on chromsome1 are depicted. NPC data in blue and MEF data in red. The ToR Y axis represents normalized log_10_ of S\G. Arrows point to examples where the changes in the ToR between MEF and NPC are correlated with changes in the LADs between these 2 tissues. Note the nearly perfect anti-correlation between the ToR and LADs in the corresponding tissue.

Measuring the ToR at multiple mouse tissue types also allowed us to study changes in the ToR and their relations to changes in other global genomic features. As mentioned before one such global feature is the association of the DNA with the lamina. Recently, the Lamina-Associated Domains (LADs) were determined in four different mouse tissue types – ES cells, NPC, AS and MEF [22]. Comparison between our ToR data in two of these tissue types (NPC and MEF) revealed that in both cases the most significant negative-correlation between the LAD and the ToR data is found for the corresponding tissue sample (Table 1), suggesting that changes in ToR are correlated with changes in the nuclear positioning of the region. Indeed, changes in the ToR between NPCs and MEFs, are significantly correlated with changes in LAD (R = −0.22; Figure 3).

We also analyzed the association between ToR and a list of distinct chromatin states that are associated with distinct genomic features [23]. We found that early replication is enriched for promoter and transcription states whereas late replication is enriched for genomic regions characterized by the repressive and repetitive states both in human lymphoblasts and fibroblasts (Figure S6). A similar association between early replication and open chromatin states was observed in drosophila [24].

**Table 1:** Association of Lamina-Associated Domains (LADs) with ToR.

	*ES Lamin*	*NPC Lamin*	*MEF Lamin*
ES ToR	−0.53176	−0.46859	−0.45245
NPC ToR	−0.62867	−0.60538	−0.55991
MEF ToR	−0.58786	−0.57359	−0.64432

LADs association in 3 different mouse tissues was calculated for each probe. Pearson correlation values (R) are shown between the ToR and LADs for each tissue.

**Figure 4:**
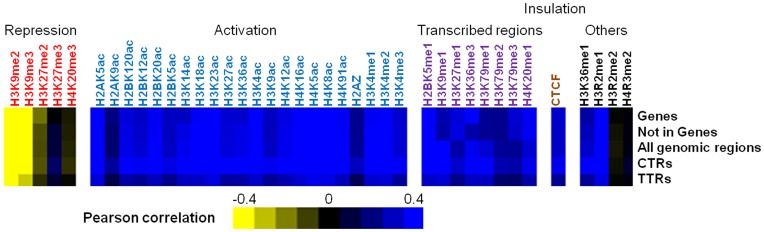
Similar correlations are found between ToR and histone marks in genes regions, intergenic regions, CTRs and TTRs. Pearson correlation coefficients between ToR and each histone mark are shown as a heat map (for numeric values see table S5). The different histone marks are grouped in light of previous reports [49]. We note that similar correlation levels are observed for TTRs and CTRs (see Discussion).

Measuring the ToR of human lymphoblasts allows direct comparison between the ToR and each individual chromatin mark measured in Cd4+ T cells [25], [26]. Due to the strong association between gene activity and chromatin structure, we decided to separately analyze the correlation between the ToR and the abundance of the chromatin marks in gene regions and in intergenic regions (see Methods). As expected, for gene regions we found negative correlation with the major repressive marks (H3K27me2, H3K9Me2 and H3K9Me3) and positive correlation with most other marks (Figure 4). Interestingly, most of those correlations were similar in intergenic regions, suggesting that those correlations are not dependent on transcription.

What causes the association between ToR and chromatin? One possibility is that chromatin structure affects the activation time of origins [27], [28]. On the other hand, chromatin structure can be a consequence of the time of replication since during replication the DNA is re-packaged and at different stages in S available chromatin building blocks may differ [29], [30]. In CTRs, this question is hard to address, due to the coupling between the time origins are activated and the replication time of the region. On the other hand, in TTRs, that either lack active origins or in which origin activation is determined by a process triggered at a distant locus [13], [31], the time of replication is mainly a function of the distance from an origin that is located in a nearby CTR. Our approach allows the systematic detection of TTRs and is therefore an ideal one for addressing this question. Indeed, we found significant correlation between the ToR and most chromatin marks in TTRs (Figure 4; note same direction of the correlation but slightly smaller magnitude), suggesting that ToR influences the determination of chromatin structure.

### Characterization of the TTRs

What are the factors that prevent independent origin firing in the TTRs? One possibility is that the TTRs are packed in a unique chromatin structure or localized in repressive nuclear compartments that prevent direct origin activation. We assessed this latter hypothesis by comparing chromatin features between CTRs and TTRs. In order to avoid biases that stem from the different ToR distribution associated with each replication activity type (Figure S7) we have selected pairs of probes from TTRs and CTRs with similar ToR. Those pairs were selected such that each probe from a CTR will have a matched (same ToR) probe from a TTR, taking care that the entire ToR range will be represented in the selected pairs (Figures S8 and S9; see Methods). Differences between these sets of paired genomic loci should be attributed to differences in the replication activity type (CTR versus TTR) and not to ToR differences.

We found no differences between such paired regions with respect to the GC content, gene density, transcription levels and association with most acetylation histone marks. On the other hand, we did find very significant association of TTRs with several chromatin features indicative of compact chromatin environment, including **i**) repressive chromatin marks H3K9Me2 (P = 10^−13^; paired t-test,) and H3K9Me3 (P = 3*10^−5^; paired t-test); **ii**) association with the nuclear envelope (P = 0.04; P = 5*10^−9^ and P = 0.003 paired t-test in MEF, ES cells and NPC respectively) and **iii**) association with the repressive nuclear compartment (Compartment B in the HiC data; P<10^−11^; paired t-test) (Figure S10). These results suggest that TTRs are located in less accessible nuclear compartments which may contribute to origin silencing or to interference with and delay of the propagation of the ToR signal.

To further investigate genomic properties that may be affected by replication activity type, we tested the tendency of transcribed regions to reside in each of the two replication activity types. We worked with two sets of transcribed regions: i) a set of expressed genes for each tissue type, and ii) transcribed exons of lincRNAs (long intergenic non coding RNAs). We compared the percentage of regions that reside in CTRs to that of 100 random sets of regions with similar distributions of lengths and of ToRs (See Methods). We found that lincRNA exons in all human and mouse tissue types have a tendency of varying magnitude, depending on tissue type, to reside in CTRs (Figure S11). This is also true for mRNA genes in most tissue types (except for human Fibroblasts and mouse NPC, Figure S12). The difference between tissues may be due to the fact that the presence of expressed genes is only one of the factors affecting the replication activity type, and can have different weight in different tissues, as compared to the effect of other factors.

## Discussion

### ToR Determination Algorithm

We introduce and describe ARTO, an algorithm that automatically assigns to each genomic region a ToR and classifies it as a TTR versus CTR. We note that classifying regions in this manner is only one possible approach to ToR activity type. A more general approach would have considered non infinite rates in the fast range as well as possibly smooth curve approximations rather than the strict piecewise linear assumption. We note that in practice considering a full range of rates would still lead to some binary partition of the data to support further analyses steps. For computational simplicity, we chose the partition as described in the method section. TTRs were identified previously [4], [6], [7], [9], [11] but only one previous study [13] describes a principled algorithmic approach to the genome-wide determination of replication activity type. This alternative method makes use of interpolated data and of inferred replication speeds. This inference is dependent on the scale of the interpolation and the authors discuss this dependence. ARTO is the only software implementation of ToR and activity type determination that is freely available to the community.

Simulations to assess ARTO’s performance using synthetic data led us to confidently determine CTR and TTR status in regions that reside >120 Kb and >180 Kb from their boundaries, respectively (Figure 1).

### Replication Activity Type Organization

We have measured genome-wide ToR profiles in four different mouse tissues and two different human tissues and applied our algorithm to analyze the data. Our systematic approach revealed that large portion of the genome is replicated as TTRs ranging from 13% in mouse lymphocytes to 37% in mouse fibroblasts. These data confirm our previous results [9] and expand them to multiple tissues. Taken together our TTR mapping results imply that large portions of the genome are replicated as TTRs, and lead to the characterization of the differences between the two organizational types of replication.

In light of recent ToR literature, there are two major interpretations of TTRs – one views them as regions lacking active origins, the other views them as regions where origin activation signals propagate along the region. Maya-Mendoza et al., [31] designate these interpretations as extending and secondary activation respectively. This study provides evidence using individual DNA fibers labeling of the co-existence of these two mechanisms in mammalian cells.

Lack of systematic information about origin location in mammals hampers the study of their tissue specificity. Specific observations that shed light on the role of origin activation were, however, reported in the literature. It has been shown that in the mouse immunoglobulin region a unique origin within a TTR is activated only in mature B cells [10]. On the other hand in the beta globin locus, the same origin is used to replicate the region at different times in different tissues [32]. The systematic classification of the genome into TTRs and CTRs, in several tissue types, as enabled by ARTO, allowed us to address the plasticity question in a more global manner. We found that 10–30% of the probes changed their replication organizational types (TTR versus CTR) in any pair of tissues, suggesting that the differential activation of origins may be more common in nature.

We found a strong enrichment for regions that change both their ToR and their replication activity type. While the independent activation of an origin in a TTR will almost certainly change both the ToR and the replication activity type, in CTRs, which are characterized by the firing of multiple origins, changing the ToR without affecting the replication activity type can take place only if multiple origins in the CTR are changed in a highly coordinated manner. Thus our observation that differences in ToR usually involved also a difference in the replication activity type, suggests that changes in ToR are local and only rarely affect entire CTRs (Figure S13). These results are in agreement with early observations about the cystic fibrosis locus in which only a sub region of the late replication zone becomes early in Caco-2 cells [33]. Interestingly, this suggests that CTRs may actually be composed of several smaller replication zones that in some tissue types are combined to larger zones, whereas in other tissue types each replicates at a different time. Studies of the differences in the ToR of ES cells and NPCs [4] revealed that small CTRs in the ES cells tend to consolidate into larger zones in NPCs (our data confirms this observation; Figures S1 and S14). Our comparison of non embryonic tissues also suggests that the basic replication units are small and that large CTRs are frequently composed of several such small zones.

### ToR and Chromatin Structure

Our data confirms earlier observations [2], [6] regarding the association of ToR and the global chromosomal organization. Early replication is associated with open chromatin (DNase-I, active histone modifications and compartment A in HiC data) whereas late replication is associated with compact chromatin (lamin association, H3K9 trimethylation and compartment B in the HiC data). It should be noted that these associations are independent of gene transcription since we found similar correlation in intergenic regions (Figure 4).

Previous studies were unclear regarding the association between ToR and specific histone modifications associated with compact chromatin. In ES and NPC cells no correlation was found with neither H3K27Me3 nor with H3K9Me3 [4], in HeLa cells a negative correlation with H3K27Me3 was observed in the ENCODE regions [34], whereas in lymphoblastoids a negative correlation was found (by us and by others [2]) with H3K9me2 and H3K9me3 and not with other known repressive marks (such as H3K27Me2, H3K27Me3 and H4K20Me3). The discordance between late replication and some repressive marks suggests that the association between the ToR and H3K9 methylation is not dependent on transcription. Indeed, we found that all the correlations observed between ToR and chromatin structure exist also in intergenic regions and hence they cannot be a mere consequence of transcription. Therefore there must be a direct connection between at least some of these marks and replication. Indeed, H3K9 methylation was recently demonstrated to be involved in replication regulation, and manipulation of this modification by over expressing the JMJD2A gene (H3K9 demethylase) causes a change of the ToR of a late locus to early replication [35].

Among the different marks of the chromatin structure some are constant between different cell types whereas others show high degree of plasticity between cells (reviewed in [36]). While differences of the ToR between tissues are approximately 30%, the association between those changes and the changes in other chromatin features still needs to be studied. As a first step in this direction we have analyzed the association between differences in ToR and differences in lamina proximity in three cell lines. Our results, as depicted in Figure 3, expand previous observations about the connection between ToR and nuclear position [6].

Our observation that the association between ToR and chromatin marks is not unique to transcribed regions (Figure 4) suggests that the association between these two processes is independent of transcription. It should be noted that finding the correlation of activation marks in intergenic regions is not surprising in light of a recent publication that demonstrates that all marks are represented both in genes and in intergenic regions [23].

### Replication Activity Type and Chromatin, Causality

Dividing the genome into CTRs and TTRs offers an approach to addressing the causal relationship between chromatin structure and ToR. Finding correlations between open chromatin and early replication in CTRs cannot highlight the mechanism lying behind such correlation since open chromatin can be either the cause for early origin firing, or the consequence of the replication at certain time in S. However, expanding this observation into TTRs (Figure 4) suggests that the latter model is correct – namely that ToR determines initial chromatin structure in cells, as we explain below. Earlier in the discussion we mentioned two views of TTRs – extending replication and secondary activation [31]. Under both models, in TTRs the ToR is determined solely by the distance from an independently activated origin (proximal to the early edge of the TTR). Therefore the actual ToR in TTRs is probably not coupled to a local independent activation event and thus cannot be directly influenced by the local chromatin structure. On the other hand, the availability of different chromatin building blocks in different stages in S [30] will still affect chromatin structure in TTRs. Although we have seen consistently slightly lower correlations in TTRs than in CTRs, it is possibly a consequence of the smaller sample size and the different ToR distribution between CTRs and TTRs. Indeed, accounting for these differences by analyzing 1000 regions from each structure and with similar ToR distribution abolished these differences (Figure S15).

Very little evidence exists in the literature to support either direction of chromatin and ToR causality (reviewed in [1]). Our observation, as explained above, viewed in this context contribute to this discussion. We note however, that under the secondary activation model for TTRs, one cannot rule out the possibility that chromatin or other sequence properties control the ToR signal propagation in the TTRs. We find this model of TTR to be unnecessarily complicated since it implicitly assumes a very tight ordering of chromatin (or sequence) codes that dictate ToR along TTRs. On the other hand, according to our interpretation, the observed ordering of the chromatin along TTRs [13], [37] is not controlled independently of the ToR but rather a consequence of its propagation. TTRs are regions that either lack active origins or in which origin activation is not directly locally controlled; however, according to both models, they probably contain latent origins that can be directly activated in different cells and during replication stress. Indeed one example of such origin is known in the immunoglobulin heavy chain (Igh) locus, in which an origin is latent in pro B cells and becomes active in mature B cells [10]. What are the factors that prevent such an origin from independently firing? A recent study has checked this question directly by systematically modifying the genetic and epigenetic status of the endogenous Igh TTR. They found that neither promoting transcription nor elevating the H3 acetylation and H3K4me3 were sufficient for activating the latent origin [38]. We report a comparison of chromatin structure between TTRs and CTRs, which revealed that the TTRs are enriched with repressive marks (H3K9 bi and tri methylation) and that TTRs are often localized in repressive compartments (nuclear envelope and HiC compartment B). These results raise the possibility that latent origin activation can be gained by removing repressive marks rather than by elevating active marks. Further experiments are needed to confirm this hypothesis.

Our results that point to correlation of chromatin marks to ToR in TTRs provide significant support to the direct effect ToR may have on shaping the chromatin structure. On the other hand the enrichment of H3K9Me2 and H3K9Me3 in TTRs supports an opposite model in which the compact chromatin prevents origin activation and they remain dormant. This finding is related to the results of a recent study [35] that points to a causal effect of H3K9 demethylase on ToR. Another study [27], [28] demonstrates a similar mechanism in which manipulating of the chromatin structure around an origin affects its activation time. It is therefore likely that there is a true mutual causal relationship between ToR and chromatin. Namely - the chromatin structure around origins plays a role in determining their activation timing thus determining the ToR of the region. The ToR affects the default packaging of the entire region which is possibly further modified, later in the life of the cell, by histone modifying enzymes that are recruited to the locus by site specific DNA binding proteins.

### Conclusion

Taken together our ability to systematically identify the replication activity type as CTR versus TTR, revealed several important features of the genome organization. First, we have shown the plasticity of the replication activity type. Second, we provided evidence for the involvement of the ToR in shaping the initial chromatin structure of the cell. Finally, comparison of ToR matched TTRs and CTRs revealed certain chromatin marks that may lead to origin silencing in the TTR regions. Therefore, our results suggest interplay between structure and ToR where ToR determines initial chromosome structure and the latter, which may change during the cell life cycle, affects ToR in the next replication round. Further developments in genomic methodologies for the direct identification of active origins are needed for direct assessment of origin activity in different genomic regions, in different cell types and in different biological conditions.

## Methods

### Cell Culture

R1 ES cells (ATCC) were cultured and differentiated into neuronal progenitor cells (NPCs) as previously described [39].

### ToR Measurements

Was performed using the DNA content methodology as described [3]. Briefly, G1 and S phase cells were extracted from each cell line using FACS sorting and the DNA from each fraction was labeled and hybridized to human or mouse custom designed Agilent DNA microarrays. Both microarrays covered the whole mouse or human genome with an average spacing of 38 Kb–50 kb, respectively. In addition, both arrays contain one chromosome (chr19 in mouse and chromosome 22 in human) printed at higher resolution (a probe every 1 kb). The replication data of mouse and human fibroblasts and lymphoblasts were already published by us [3] and the mouse ES and NPC data is new (deposited in GEO GSE17236).

### ToR Determination and Data Analysis

We developed and used the ARTO algorithm; see full description of the algorithm as well as of its performance evaluation in Supplementary Methods.

### Simulated Data

We produced 100 synthetic signals, each is 3200 samples long, that are as similar as possible to the expected ToR signal in terms of lengths of segments, slopes of lines, signal range, distance between samples etc. To these signals we added white Gaussian noise with different values of standard deviation –2%, 5%, 10%, 15% and 20% of the synthetic signal range.

### Estimating Noise in Raw ToR Data

We estimated the standard deviation of the noise component of the ToR raw data by using the robust median absolute deviation (MAD) estimator (see Supplementary Methods). We found that the standard deviation of the noise in the raw data of most samples ranges between 14% and 22% of the raw data range, with the exception of one sample of L1210 which had a higher noise level (∼30% of the raw data range).

### Replication Activity Type Classification

To analyze the confidence in the assignment of each probe to a replication activity type (CTR versus TTR), we calculated the percentage of correct assignment in the simulated data as a function of the distance from the segment ends. This was done separately for CTRs and for TTRs. We found (Figure S16) that for CTRs we can determine the replication activity type with 80% certainty in regions distant more than 3 simulated probes (120 KB) from the segment ends. In TTRs this distance grows to ∼4.5 probes (180 KB). These distances were used when applying ARTO to the actual measurement data in order to determine the replication activity type at every genomic locus. Regions that are closer to segment ends were left as undefined (see Figure 1).

### Determining ToR and Replication Activity Type of Genomic Regions

As described before, our algorithm outputs an estimate of ToR and an assignment to a replication activity type for each genomic locus where a probe is present. However, for some analyses we would like to determine ToR and replication activity type assignment for genomic regions, such as genes, lincRNA exons and regions of specific chromatin state. Genomic regions may not have the same ToR through the entire region, and they might also reside in more than one segment, and moreover – reside both in a TTR and in a CTR. In order to determine ToR, we investigated the difference between averaging the ToR in the start and end points of genes and taking the ToR in their middle, and found that in less than 1% of the genes there was a difference of over than 10% of the S-phase length. We also found that only around 5% of the genes reside in more than one segment. Therefore, the ToR of a genomic region was set to be the ToR of its middle point. To determine the replications structure of a genomic region we first check which segment contains the middle point of the region. If at least part of the region of interest is not in an undefined part of this segment, we give it the segment’s replication activity type assignment. Otherwise the region is undefined.

### Selecting Paired Probes from CTRs and TTRs

In order to avoid biases originating from the different ToR distribution of CTRs and TTRs we selected, in each tissue type, 600–1000 pairs. Each pair contains one probe from a CTR and another form a TTR with almost identical ToR. This pairing was obtained by randomly drawing a ToR value using a uniform distribution, and selecting two probes with the closest ToR as possible, one in CTR and the other in TTR. Each probe from one set will therefore have a matching probe from the other set with the same ToR. The final distribution of ToR for both sets is very similar, and close to being uniform (Figure S6). To find intergenic pairs of probes we have looked only at probes which are located more than 20 Kb away from genes. To find pairs of probes within genes, we determined for each gene its ToR and whether it is in a CTR or a TTR, and then continued with the random selection described above. This general scheme was applied to get the following sets of probes:

- A set of 1000 intergenic pairs of probes in Molt4 (human lymphoblasts) that was used for the analyses of the HiC data, 39 chromatin modifications and 51 chromatin states.
- Three sets of 600 intergenic pairs of probes in mouse ES, NPC and MEF (one set for each tissue type) that were used for the Lamin analysis (see table S4 for complete lists of probes used for these analyses).

### Genes and lincRNAs Residing in CTRs

Expression was downloaded from the following sources - mouse ES, NPC and MEF expression from [40]; Foreskin fibroblasts expression (GSE5416) from [41] and MOLT4 expression (GSE6495; average of three replicates) from [42]. The L1210 expression data was generated by us previously [9]. Expressed genes were taken to be genes with expression values higher than a certain threshold, which was set for each tissue separately: for mouse tissues we used a threshold of 10, for human lymphoblasts –5, and for human fibroblasts we used a threshold of 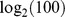. The lists of lincRNA exons in human and mouse tissue types were taken from [43], [44], [45], and filtered using a maximal independent set algorithm to contain only non-overlapping exons. There is no sufficient tissue specific information for lincRNAs, and we therefore used all lincRNAs data in our analysis.

For each gene we determined its ToR and replication activity type as described above, and filtered out genes that are assigned to an undefined replication region. For each tissue type we calculated the percentage of genes residing in CTRs. To obtain random control we computed the percentage of CTRs in every set out of 100 sets of randomly drawn regions that have similar properties as the set of genes – reside in the same chromosomes as the genes, with the same length as the genes and similar ToR, and are not assigned to an undefined replication region. The analysis of lincRNAs was performed in the same manner.

### Analysis of Chromatin Structure

Histone modifications data of the human CD4+ blood cells [25] was compared to the ToR Molt4 data. The chromatin marks density for each of the ToR probes was calculated as the number of sequence reads in a window of 30 kb around the probe. The data of the Lamina-Associated Domains (LADs) was compared to ToR data of a relevant tissue. [46]. The fibroblasts LAD data [46] was correlated to the FFT ToR data, and the mouse LAD data [22] was used for comparison with mouse ES, NPC and MEF ToR data. The DNase-I hyper sensitive sites data of CD4+ human cells [47] was compared to the human MOLT4 ToR data. Long-range chromatin interactions (Hi-C) in human blood cells data was compared to the human Molt-4 ToR data. For each probe we used the eigen values reported in [48] as an estimators for a region to be in the open (A) or compact (B) genomic compartments. The genome-wide methylation data in human primary fibroblasts [18] was compared to the FFT ToR data. For each probe, the methylation percentage in a window of 5 kb surrounding the probe was calculated. In all cases Pearson correlations were calculated for all probes, separately for probes residing in genes and in intergenic regions (>20 Kb from genes) and for probes residing in CTRs and in TTRs.

## Supporting Information

Data Analysis Supplement S1 (DOCX)

Figure S1 **ToR Autocorrelation.** Autocorrelation was calculated for all probes on chromosome 1 (after sorting) before and after segmentation (dashed and solid lines respectively). The probe spacing was on average 40 kb. A significant autocorrelation is observed for all tissues at least until lag = 100. Note that the autocorrelation is the smallest for the ES cells (and also NPC1 which resembles ES cells, see also S3) which suggests smaller replication zones in these cells.
(TIF)

Figure S2 **Correlation between ToR, genomic features and expression.** Representative plots of the ToR against GC content (left panels) and gene density (middle panel) are shown. The cell type (ES, upper row, NPC middle rows and L1210, bottom rows) and the Pearson correlation coefficient are written above each graph. The right plots shows the difference in the expression distribution between early (cyan) and late (blue) genes. The statistical significance of the difference between the distributions (Student T test) are written above each plot. Note that the correlation between ToR and GC content/gene density is high and significant in all cell types, however, in stem cells the correlation is the lowest.
(TIF)

Figure S3 **The effect of relaxing fork rate assumption on ARTO results.** We tested ARTO using two different fork rate ranges, namely 0.25–4 Kb/minute (A top panel), and 0.25–10 Kb/minute (A bottom panel). B shows the distribution of differences in the results. Note that for >80% of the probes inferred ToR was affected by less than 5%. Our test also shows that 4.5% of probes changed from CTR to TTR and no probe changed from TTR to CTR.
(TIF)

Figure S4 **ToR Correlation before and after segmentation.** Up-left: correlation of raw ToR measurements between mouse tissues. Up-right: correlation of segmented ToR between mouse tissues. Down-left: correlation of raw ToR measurements between human tissues. Down-right: correlation of segmented ToR between human tissues. As expected, the correlations between replicates of the same tissue are higher than the correlation between tissues (except in mouse NPC). The correlation is greatly improved after segmentation.
(TIF)

Figure S5 **Comparison of replication activity type between tissues.** Percentage of probes with identical replication activity type assignment (CTR or TTR) for each pair of mouse (on the left) and human (on the right) tissues. Replicates of the same tissue show a higher percentage of identical assignments.
(TIF)

Figure S6 **ToR distribution in 15 chromatin states.** The average ToR (S/G1 log ratio) in the regions associated with each of the 15 distinct chromatin states in lymphoblasts (blue) and fibriblasts (red) published by Ernst et al. (Nature 473, 43–49, 2011). Early replication is enriched for promoter and transcription states, whereas late replication is enriched for genomic regions characterized by the repressive and repetitive states.
(TIF)

Figure S7 **comparison of ToR distribution between CTRs and TTRs.** ToR distribution of all the probes in CTRs (upper panel) and TTRs (lower panel), in human lymphoblasts. The ToR distribution is very different between the two types of regions – most CTRs replicate early or late, while TTR ToR is mostly in middle S-phase.
(TIF)

Figure S8 **comparison of ToR distribution in the paired probes of CTRs and TTRs.** ToR of 1000 paired intergenic probes in CTRs (upper panel) and TTRs (lower panel), in human lymphoblasts. The probes were randomly selected so that the ToR distribution of both sets of probes will be the same and close to uniform.
(TIF)

Figure S9 **ToR of paired probes.** Scatter plot of the ToR in 1000 paired intergenic probes in CTRs (x-axis) and TTRs (y-axis), in human lymphoblasts. Each pair of matched probes has the same ToR.
(TIF)

Figure S10 **CTRs and TTRs correlation with chromatin structure.** Scatter plots representing the correlations between the ToR and various chromatin features are shown for 1000 probes residing in CTRs (blue) and TTRs (red) regions with matched ToR. In all plots, x-axis represents ToR. Note that the ToR is positively correlated for the HiC data (In which higher values are for open chromatin) and negatively correlated with the other repressive markers both in TTRs and CTRs. However the CTRs values are consistently lower for repressive markers and higher for activation markers suggesting that TTRs are packed in closer chromatin than CTRs.
(TIF)

Figure S11 **LincRNA transcribed exons have a tendency to reside in CTRs.** For each tissue type, the percentage of lincRNA transcribed exons that reside in CTRs is marked in red. Histograms of the percentage of randomly drawn genomic regions (with similar properties as lincRNA exons) assigned to CTRs in 100 random sets are marked with blue. In all mouse and human tissues, lincRNA transcribed exons have a tendency to reside in CTRs, more than would be expected from the random control.
(TIF)

Figure S12 **Expressed genes have a tendency to reside in CTRs.** For each tissue type, the percentage of expressed genes that reside in CTRs is marked in red. Histograms of the percentage of randomly drawn genomic regions (with similar properties as lithe genes) assigned to CTRs in 100 random sets are marked with blue. In most tissues (except for human fibroblasls and mouse NPC) expressed genes have a tendency to reside in CTRs, more than would be expected from the random control.
(TIF)

Figure S13 **Associations between changes in the ToR and in the replication activity type.** Schematic representations of ToR maps in two regions in which the ToR is different between two tissues (red and blue). The two regions differ in the extent of ToR change (in A the change of the ToR affects an entire CTR whereas in B it was limited to a small portion of the CTR). The dotted lines separate sub regions according to their replication activity type. The letters below the graphs indicate for each segment whether its ToR and its structure was constant (c) or variable (v) between the two tissues. Note that in B most of the regions that changed their ToR also changed their structure whereas in A ToR change with constant structure is much more common.
(TIF)

Figure S14 **Distribution of CTR segments lengths in mouse tissues.** The distribution of CTR segments lengths (in bp) for all four mouse tissue types, as well as of cCTRs (constant CTRs, which are regions that are defined as CTRs in all four mouse tissues). In each histogram, the percentage of regions longer than 1 Mb is written.
(TIF)

Figure S15 **Similar correlations are found between ToR and histone modifications in CTRs and TTRs.** The analysis shown in Figure 4 was repeated for 1000 TTRs (black) and 1000 CTRs (grey) with matched ToR. the Pearson correlation coefficients are shown for each histone modification. Note that the small differences between the CTRs and TTRs seen in Figure 4 disappear when both groups were on the same size (1000 regions) and the same ToR distributions.
(TIF)

Figure S16 **Confidence level in CTR and TTR assignments vs. distance from segment ends.** The fraction of correctly assigned probes as a function of distance from segment ends (measured in probes) in simulated data, reflecting the confidence level of replication activity type assignment for both CTR (A) and TTR (B). The averaging is done on different segments length, for example for CTRs: all segments and segments with lengths over 30, 60 and 90 probes.
(TIF)

Table S1 **Mouse ToR data.**
(XLSX)

Table S2 **Human ToR data.**
(XLSX)

Table S3 **Comparison of the replication activity type between tissues.**
(TIF)

Table S4 **Complete lists of paired probes used in the analyses.**
(XLSX)

Table S5 **Numeric values of** **Figure 4** **.**
(XLSX)

Table S6 **Parameters optimal value vs. amount of noise.**
(TIF)

Table S7 **Comparison between L1210 replicates before and after segmentation.**
(TIF)

Table S8 **Comparison between MEF replicates before and after segmentation.**
(TIF)
