# Supplementary material for: Systematic Determination of Replication Activity Type Highlights Interconnections between Replication, Chromatin Structure and Nuclear Localization
Source: PLoS One. 2012 Nov 7;7(11):e48986. doi: 10.1371/journal.pone.0048986 (PMC3492150; doi:10.1371/journal.pone.0048986)
Supplement: Data Analysis Supplement S1 — (DOCX) [file pone.0048986.s001.docx]

**Systematic determination of replication activity type highlights interconnections between replication, chromatin structure and nuclear localization**

**Supplementary Methods**

In this supplement we address data analysis aspects of the paper, as follows:

- Section 1: Description of the ToR and replication activity type determination algorithm, which we call ARTO.
- Section 2: Performance of ARTO.
- Section 3: Statistical methods that were used to support the biological findings described in the paper.

1. **ARTO algorithm**

Our ToR data is very similar to the data acquired using aCGH arrays, since it represents a comparison between two cell populations. The literature proposes several methods for the analysis of aCGH data that perform segmentation in various ways in order to find variations in copy number (Lipson, Aumann, Ben-Dor, Linial, & Yakhini, 2006; Ben-Yaacov & Eldar, 2008; Olshen, Venkatraman, Lucito, & Wigler, 2004). These segmentation methods assume a piecewise constant model, as they seek aberrations (deletions or amplifications) in the signal. However, the ToR signal is composed of linear segments that correspond to TTRs, in addition to the constant segments that correspond to CTRs. Current aCGH segmentation methods do not fit this model, and therefore we developed a novel algorithmic approach to address this segmentation problem. Our algorithm, ARTO, is available in Matlab implementation at http://bioinfo.cs.technion.ac.il/people/zohar/ARTO/.

- 1. **Algorithm assumptions**

Our raw data can be viewed as noisy samples of a signal, each sample $y_{i}$ representing the ToR of a specific genomic location in the DNA $x_{i}$. As demonstrated in the literature (Farkash-Amar, et al., 2008; Hiratani, et al., 2008) there are two types of genomic regions: replication zones containing active origins firing at the same time and are therefore constant ToR regions (CTRs), and temporal transition regions (TTRs). This gives rise to two types of segments in the ToR signal: the CTRs will appear as constant ToR regions, while the TTRs will be represented by linear lines with a range of slopes that correspond to the replication fork-rate. In addition, since in most cases replication stops when replication forks meet, the ToR signal will rarely have discontinuities. We therefore expect the ToR signal to be a piecewise-linear continuous signal. This principle forms the basis of our signal processing algorithmic approach.

Our algorithm takes raw ToR measurement signals and for each genomic location results in:

- An estimate of the ToR.
- An assignment of the replication activity type, namely – CTR or TTR.
  1. **Algorithm Description**

The goal of our algorithm is to reconstruct the ToR signal $t\left(x\right)$ from noisy per-probe measurement results, under the assumptions stated above, using the following general approach (described in detail in the next subsections):

- Pre-processing steps that include noise filtering and parameter setting (see Subsection 1.2.1).
- Divide the signal into overlapping windows with a constant number of samples $W$. For each window:
  - Using the Hough transform (Hough, 1962; Illingworth & Kittler, 1988), search for potential lines that match some of the points in the window and have a slope within a given range (see Subsection 1.2.2).
  - Based on these lines compute an approximate best-fit continuous segmentation for the window using dynamic programming (see Subsection 1.2.3).
- Start the next window at the last computed breakpoint of the current window, until reaching the last sample.

Figure 1 in the main paper shows an example of the segmentation results on both synthetic and real data.

- - 1. **Pre-processing steps**

Before approaching the segmentation itself, we pre-process the data in order to eliminate noise and outliers. First, we estimate the standard deviation of the noise component of the signal by using the robust median absolute deviation (MAD) estimator, described by Donoho (Donoho, 1995) and used in HaarSeg (Ben-Yaacov & Eldar, 2008):

$$\hat{\sigma}=\frac{median\left(\left|y_{n}-y_{n-1}\right|/\sqrt{2}\right)}{0.6745}$$

This estimate is used in setting different parameters in the algorithm as well as in performing the Hough transform, as will be described in the next subsections. We also apply a moving average to the signal, with a window of 5 samples in order to smooth out the noise.

- - 1. **Hough Transform**

The Hough transform is a feature extraction technique used in computer vision in which the problem of finding parametric curves in the data space is converted to finding local maxima in the parameter space (Hough, 1962; Illingworth & Kittler, 1988; Duda & Hart, 1972; Ballard, 1981). To extract straight lines the data points are usually transformed to the $\left(\rho,\theta\right)$ plane, where $\rho$ is the length and $\theta$ is the orientation of the normal vector to the line from the origin (Figure S17). In the standard Hough transform, a matrix representing the $\left(\rho,\theta\right)$ plane with a certain resolution is computed in the following manner. For each data point $\left(x_{i},y_{i}\right)$ all pairs of angles $\theta$ and lengths $\rho$ representing lines passing through it are calculated using:

$$\rho=x_{i}\cos\left(\theta\right)+y_{i}\sin\left(\theta\right)$$

and the appropriate $\left(\rho,\theta\right)$ entries in the matrix are accumulated. After this accumulation of votes from all data points, the local maxima, which represent the line segments prevalent in the data, are found.

Many variations to the standard transform were suggested in the signal processing literature. One suggested variation (Thrift & Dunn, 1983) deals with noisy data by using a band (or a *voting kernel*) instead of a simple binary vote. This voting kernel is defined by the function $c\left(\rho-x_{i}\cos\left(\theta\right)-y_{i}\sin\left(\theta\right)\right)$, where $c\left(r\right)$ is continuous, symmetric and non-increasing as a function of $\left|r\right|$.

In our work we used a Hough transform with a Gaussian voting kernel to find potential linear lines in a data window, while adding some modifications to fit the described biological constraints and to address the relatively large noise component. More specifically:

- - - - 1. We calculate a voting matrix $V_{i}$ in the $\left(\rho,\theta\right)$ plane for each data point$p_{i}=\left(x_{i},y_{i}\right)$. The matrix spans the range of lengths $\rho=\left[-\rho_{\max},\rho_{\max}\right]$ such that

$$\rho_{\max}=\sqrt{\left(\max\left\{x_{i}\right\}\right)^{2}+\left(\max\left\{\left|y_{i}\right|\right\}\right)^{2}}$$

with the $\rho$ resolution matching the distance between data points and without exceeding 2000 bins. The matrices $V_{i}$ span angles $\theta$ between the angle which corresponds to the maximal fork-rate and the one which corresponds to the minimal fork-rate in 2 degrees steps, for both negative and positive angles, and a zero angle (we used a fork-rate range of 0.25-4 Kb/min based on (Farkash-Amar, et al., 2008), and then the actual range of $\theta$ depends on the S-phase length). For each angle $\theta$ in this range, a Gaussian voting kernel with width

$$\sigma\left(\theta\right)=\hat{\sigma}\sin\left(\theta\right)$$

and centered around $\left(\rho,\theta\right)$is inserted to the matrix at the appropriate column. That is:

$$V_{i}\left(r,\theta\right)=\exp\left\{-\left(r-\rho\right)^{2}/2\left[\sigma\left(\theta\right)\right]^{2}\right\}$$

Note that the width of the Gaussian is proportional to the estimated noise $\hat{\sigma}$, taking into account that the noise is only in the direction of the ToR measurement since the genomic location of the probe is known (and therefore there is no noise associated with ).

- - - - 1. Since we would like to avoid the influence of distant points which are not part of a segment on the determination of its parameters, the data points are divided into sets of neighboring points which are likely to be part of the same segment. We define$n_{\min}$ to be the minimal number of data points allowed in a segment, and use it as a parameter of the algorithm. We then apply the following iterative process:

While there is one point or more which is not part of any set:

1. Sum the voting matrices of all data points which are not part of any set, and choose the line with the highest sum of votes.
2. Consider the data point $p_{0}$ which is not part of any set and with the highest vote for this line, and then choose the line $l_{0}$ with the highest vote from this point. The reason for re-choosing a line is to eliminate the influence of distant data points, as mentioned above.
3. For each direction, starting from $p_{0}$, insert to the set all data points up to the first point $p_{k-1}$ that satisfy:

$$\frac{1}{\sqrt{k}}\sum_{i=0}^{k-1} v\left(p_{i}\right)<\frac{1}{\sqrt{k-1}}\sum_{i=0}^{k-2} v\left(p_{i}\right)<\frac{1}{\sqrt{k-2}}\sum_{i=0}^{k-3} v\left(p_{i}\right)$$

where $v\left(p_{i}\right)$ is the vote of data point $p_{i}$ for line .

- 1. If the difference of this set with a previously determined set is less than 5 points, then form a union of this set and the set that has the largest intersection with it.
  2. If the set is too small (the number of points in it is smaller than $n_{\min}$) and has a non-empty intersection with another previously determined set, then form a union of the this set and the set that has the largest intersection with it.
  3. Otherwise, add this set to the existing list of sets.

Constant lines usually have the highest sum of votes since they can be affected by data points from the entire window. For this reason, in order to find all potential lines within a window including those supported by a small number of points, the process of dividing the points into sets is performed three times – once for negative angles, once for positive angles and once for constant lines. All resulting sets (many of them overlapping) participate in the next step.

- - - - 1. For each set of points a single voting matrix is calculated as the weighted sum of the data points within the set. The weighting function is given by:

$$U\left(p_{i}\right)=\left\{\begin{matrix} \left(1+e^{-6\frac{\left(i-\left(m-1\right)/2\right)}{\left(m-1\right)/2}}\right)^{-1}\text{ }i=0,...m-1 \\ 1\text{ }i=m,...N-m-1 \\ \left(1+e^{-6\frac{\left(\left(N-1-i\right)-\left(m-1\right)/2\right)}{\left(m-1\right)/2}}\right)^{-1}\text{ }i=N-m,...N-1 \end{matrix}\right.$$

where $N$ is the number of points in the set and .

- - - - 1. Each voting matrix calculated in the previous step will now have a single maximum, representing the line supported by this set of points. The maximal point in each of the voting matrices is found, and its $(\rho,\theta)$ parameters are extracted.

Finally, after performing the modified Hough transform, the sum of votes of every one of the potential lines that was found in the above procedure is compared to a threshold $T$ so that only lines with high enough sum of votes will be passed to the next step. The threshold is given by .

- - 1. **Segmentation by Dynamic Programming**

Given all potential lines within a window, we wish to find the best segmentation in the window, composed of these lines.

To perform the segmentation of a specific data window with a set $L$ of potential lines calculated in the previous algorithm step, we first find all intersection points between all the potential lines in increasing order. Assume we have $\left(N-1\right)$ intersection points $z_{1},...z_{N-1}$ ($z_{i}$ is the x-coordinate, or the genomic coordinate, of the intersection points) and let $z_{0}$ and $z_{N}$ denote the beginning and end of the window. For each region defined by two consecutive intersection points $z_{n},z_{n+1}$ and for each line $l\in L$ we calculate a score

$s\left(n,l\right)=\sum_{p_{i}\in\left[z_{n},z_{n+1}\right]} v\left(p_{i}\right)$.

This score is the total vote of a set of data points within a region $\left[z_{n},z_{n+1}\right]$ supporting the line $l$. The overall score assigned to any candidate segmentation $S=\left[l_{1},...l_{j},...l_{N-1}\right]$ along the window is therefore:

$Score(S)=\sum_{j=1}^{N-1} s\left(j,l_{j}\right)$.

We now seek a continuous segmentation with maximum score.

For this purpose we build a $K\times N$ matrix $\Phi$, where $K$ is the maximal number of segments within a window, a parameter of the algorithm that we set to $K=12$. Each entry in the matrix $\Phi\left(k,n\right)$ should contain the maximal score of the continuous segmentation using $k$ segments until and including the region $n$. It is determined through the following dynamic programming scheme:

$$\begin{matrix} \Phi\left(1,n\right)=\max_{l\in L} \left[\sum_{i=1}^{n} s\left(i,l\right)\right] \\ \Phi\left(k,n\right)=\max_{1\leq m<n} \left\{\Phi\left(k-1,m\right)+\max_{l\in\Theta_{mk}} \left[\sum_{i=m+1}^{n} s\left(i,l\right)\right]\right\} \end{matrix}$$

where $\Theta_{mk}\subseteq L$ is the subset of lines which pass through the end-point of the segmentation up to the region $m$. After completing the calculation of the matrix we consider its last column $\Phi\left(k,N\right)$ and seek the segmentation of the entire window with the highest score. It is clear that the more segments we allow, the higher the segmentation score will be because we enable each small region to select the line closest to its associated data points. To avoid over fitting through segmentation with a large number of small segments we apply a penalty which is linear in $k$, and select the final segmentation according to:

$\max_{1\leq k\leq K} \left\{\Phi(k,N)-ck\right\}$.

We used $c=2$ as the penalty constant. Finally, we perform backtracking to find the segmentation which gives this highest score.

While a dynamic programming procedure similar to the above would have produced the optimal segmentation using the lines in $L$, the above procedure does not actually produce the optimum continuous segmentation. The continuity requirement is addressed in seeking a maximum over $\Theta_{mk}$ and not over all of $L$ in (*). By doing this we guarantee that the solution is continuous but we do not guarantee optimality. Let us assume we found $S_{m}$ - the highest score continuous segmentation until the region $m$, and we are now looking for the next segment only among the lines which intersect at the end-point of this segmentation. It is possible that another segmentation $S_{m}^{*}$ which ends at a different point and received a lower score than $S_{m}$ will have a continuing segment which, when combining their scores, will have a total score which is higher than that of $S_{m}$ and its continuing best segment. However, in most practical cases, the best segmentation is indeed found using the above dynamic programming procedure. To even further improve the results and guarantee optimality in even more cases, we save three layers of the dynamic programming matrix: $\Phi\left(k,n,j\right),\text{ j=1,2,3}$, containing the highest, second-highest and third-highest segmentation scores for each $k$ and $n$.

At the end of this step, a final segmentation of the data window is produced. To improve the estimation of the last segment in the window and to assure that all the data points supporting a line are taken into account together, we start processing the next window from the last breakpoint between segments that is found in the current window.

- 1. **Algorithm parameters**

In addition to biological conditions such as S-phase length and the expected fork-rate range which are inputs of the algorithm, two parameters have to be selected before applying the algorithm to the raw data:

Number of samples in a data window $W$.

Minimal number of data points in a segment $n_{\min}$. The algorithm enables setting a different parameter for segments representing active replication zones and those representing TTRs.

Maximal number of segments in a window $K$.

These three parameters should satisfy the condition $W<K\cdot n_{\min}$so that even if all segments in a window are $n_{\min}$-long, ARTO will still be able to detect all segments. Therefore, only two of these three parameters have to be selected before applying the algorithm to the raw data. In the results reported in the main paper we had set $K$ to a fixed value of 12. When $K$ is determined, it effectively determines how many short segments can be in the final segmentation. Therefore, lower $K$ leads to more short segments in the segmentation. Higher $n_{\min}$ means that small segments will not be detected on the one hand, but on the other hand small segments (usually representing TTRs) will not be falsely created by the segmentation. We performed simulations to validate these assumptions. Namely, we performed segmentation of synthetic signals with an increasing amount of noise and calculated RMSE and percentage incorrect assignment of replication activity type (see Section 2 below).Our simulations covered several parameters values: $W=80,120,...,320$ and $n_{\min}=2,4,...,20$. Our simulations show that as expected these two parameters should be increased along with the percentage of the noise out of the signal range (Table S6).

Other fixed parameters used by the algorithm which were mentioned in the previous sections are:

1. Number of samples averaged in the pre-processing step. This parameter is set to 5.
2. $K$ , Representing the highest number of segments per window, set to 12..
3. Penalty constant $c$ in the dynamic programming step. This parameter is set to 2.

We have used simulations with synthetic data to determine some of the parameters used in practice. A full description of these simulations is available in YD’s thesis on the Technion website.

1. **Algorithm performance**

To evaluate the accuracy of the ToR signal reconstruction we have used both synthetic data and real data from technical repeats. We calculated two error measures:

1. To estimate the error in determining ToR we used Root Mean Square Error (RMSE), computed by:

$$RMSE=\sqrt{\frac{1}{N}\sum_{i=1}^{N} \left(\hat{t}_{i}-t_{i}\right)^{2}}$$

where $N$ is the number of probes, $t_{i}$ is the value of the synthetic ToR signal at a probe $i$ and $\hat{t}_{i}$ is the ToR assigned to the same probe by ARTO.

- To estimate the error in association to replication zones we calculated the percentage of different associations for the same location between the reference signal and the segmentation result.
  1. **Simulated data**

We produced 100 synthetic signals, each is 3200 samples long, that are as similar as possible to the expected ToR signal in terms of segments length, lines slopes, signal range, distance between samples etc. To these signals we added white Gaussian noise with different values of standard deviation – 2%, 5%, 10%, 15% and 20% out of the synthetic signal range. We then performed segmentation and compared the results to the original synthetic signals using the two error measures described above. In addition we compared between the segmentation results of the same synthetic signal with two different noise components with the same standard deviation. The results are shown in Figure S18. As expected, both error measures increase with the increase of noise. While the RMSE seems to grow linearly with the amount of noise, the error in replication activity type assignment has a negative curvature growth.

To further analyze the confidence in the association of each probe to a type of replication zone, we calculated the percentage of correct associations in the simulated data as a function of the distance from the segment ends. We did this separately for CTRs and for TTRs, and for segments with different lengths (Figure S16). We found that for CTRs we can determine the replication activity type with 80% certainty in a distance of 3 simulated probes (approximately 120KB) from the segment ends, whereas in TTRs this distance grows to ~4.5 probes (180KB). We used this analysis and determined the association to replication zones in the real ToR data only for genomic locations that are in a distance from segment ends which exceeds these thresholds (see Figure 1, where undefined regions are marked with gray, and CTRs and TTRs are marked with black).

To improve segmentation results, we would like to combine the replicates of the same tissue type, since their underlying ToR signal is expected to be the same. We do this by simply performing the segmentation on two replicates together (and therefore the input to the algorithm is a double amount of data points). We tested this scheme using two noisy signals originating from the same synthetic signal, and compared the segmentation result of each of them separately as well as of both of them together with the original synthetic signal. As can be seen in Figure S18, we found that as expected, the RMSE and the error in replication activity type assignment are both lower when taking the two replicates together. We therefore used this approach when performing segmentation of the real ToR data.

- 1. **Real data of technical repeats**

Out of the real raw ToR data, the measurements in two of the tissues – mouse L1210 and MEF - were technical repetitions of one another. To perform qualitative evaluation of the algorithm performance we used the segmentation results of these technical repeats. We calculated the correlation between repeats of both the raw data and the segmented data, and we compared the association to replication zone of all probes (Tables S7, S8).

For the two L1210 repeats, there was a high correlation of the raw data ($R=0.9$) which was then improved by the segmentation to reach $R=0.98$ in the segmented data. The association with replication zone was practically the same between the two replicates – 99.3% of the probes were associated to the same type of replication zone. Among all tissues examined in our work, L1210 had the highest correlation between replicates before and after segmentation, and the best fit in replication activity type, demonstrating the quality of the algorithm results.

Though the 4 MEF repeats showed a lower correlation before segmentation (correlation between pairs ranges between 0.55 and 0.73), the correlation coefficients after segmentation were much higher (0.93 to 0.97) and are the second highest among the examined tissues. 90% to 94% of the probes in each pair of replicates were associated with the same type of replication zone.

- 1. **Running Time**

We measured ARTO running time on each chromosome for each of the replicates in the real data, as well as for replicates of the same tissue type taken together. We plotted the measured running time as a function of the number of probes in the data (Figure S19). We found that the growth in running time is linear with the number of data points, or probes, in the algorithm input.

1. **Statistical methods**
   1. **Decoupling ToR and replication activity type**

ToR is highly correlated with the association to an active replication zone (CTR) or to a TTR. For example, most of the early replicated regions are active replication zones, while middle S-phase replication usually occurs in TTRs. When wishing to assess the correlation of a specific genomic property with replication activity type, we needed to decouple this very tight relationship. We therefore selected randomly two sets of probes – one in CTRs and one in TTRs, so that each probe from one set will have a matching probe from the other set and so that the ToRs of these members of the matched pair are the same. The statistical analysis is then done on these two sets of probes that have the same ToR distribution, which is close to uniform.

- 1. **Tendency of a set of genomic regions to reside in a specific type of replication region**

To understand whether genes (or any other set of genomic regions) have a tendency to reside in CTRs or in TTRs, we would like to check if a similar set of regions selected randomly will act the same. We randomly drew regions with similar properties – reside in the same chromosome, with the same length and similar ToR, and repeated this 100 times. We then compared the percentage of regions that reside in CTRs or in TTRs out of the set of interest to that of each of the randomly selected set, as well as to their mean and standard deviation. Finally, we calculated an empirical p-value as:

$$p=\frac{1}{100}\left(\begin{matrix} \text{\# of random sets for which the \% of regions in CTRs or in TTRs} \\ \text{is }\geq\text{ than \% in actual real set of interest} \end{matrix}\right)$$

**Figures**


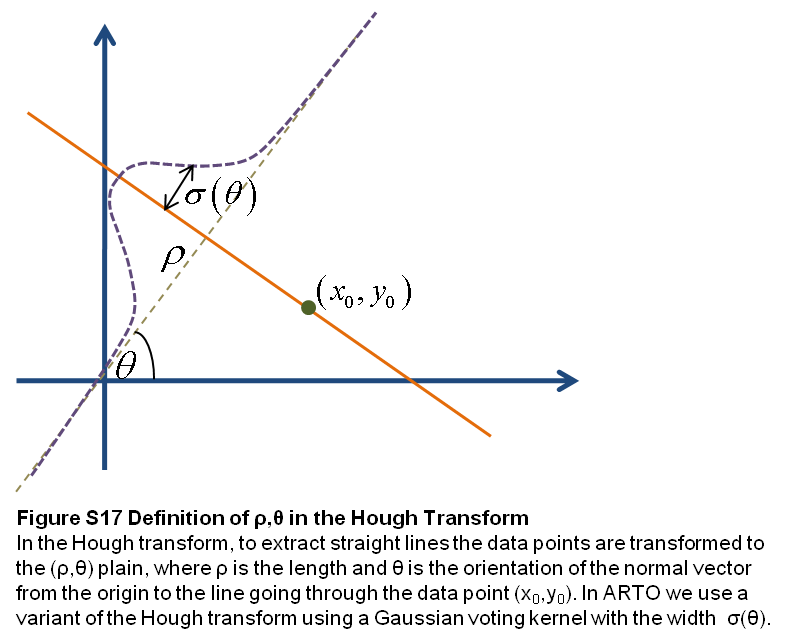


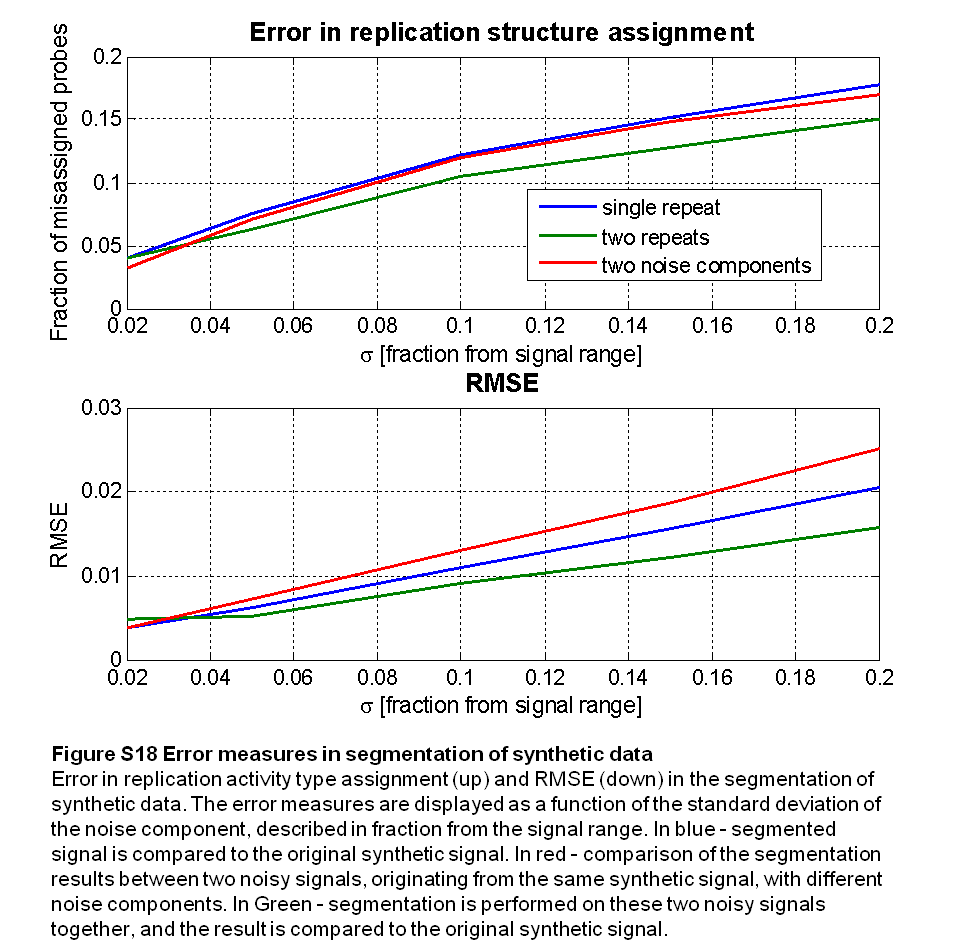


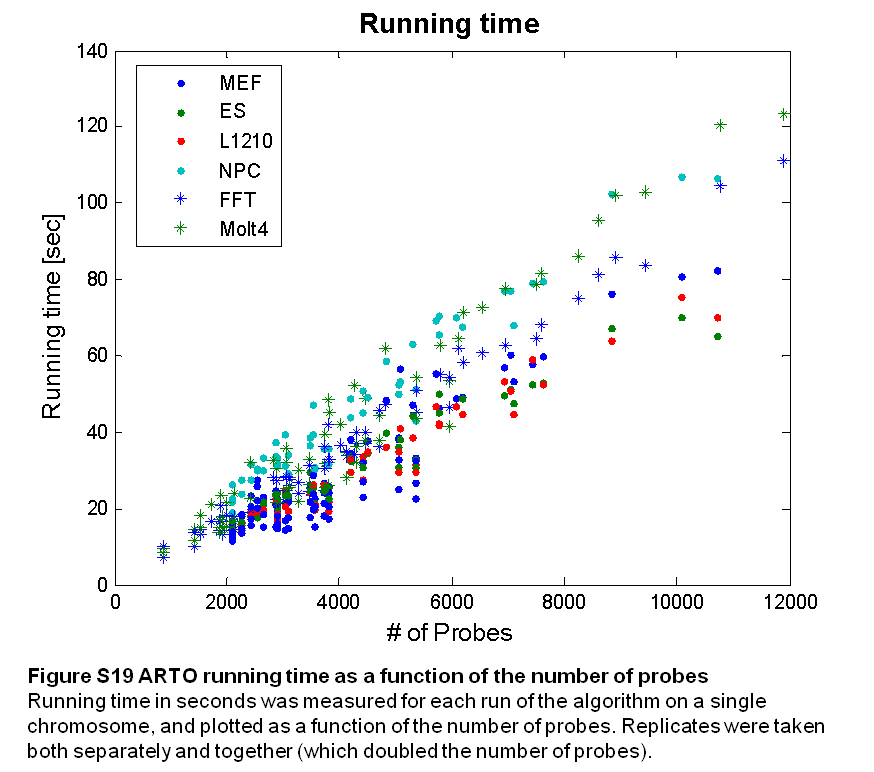


# Bibliography

Ballard, D. H. (1981). Generalizing the Hough Transform to detect arbitrary shapes. *Pattern Recognition* *, 13* (2), 111-122.

Ben-Yaacov, E., & Eldar, Y. C. (2008). A fast and flexible method for the segmentation of aCGH data. *Bioinformatics* *, 24* (16), i139-i145.

Donoho, D. L. (1995). De-noising by soft-thresholding. *IEEE Transactions on Information Theory* *, 41* (3), 613-621.

Duda, R. O., & Hart, P. E. (1972). Use of the Hough Transform to detect lines and curves in pictures. *Communications of the ACM* *, 15* (1), 11-15.

Farkash-Amar, S., Lipson, D., Polten, A., Goren, A., Helstetter, C., Yakhini, Z., et al. (2008). Global organization of replication time zones of the mouse genome. *Genome Research* *, 18*, 1562-70.

Hiratani, I., Ryba, T., Itoh, M., Yokochi, T., Schwaiger, M., Chang, C. W., et al. (2008). Global reorganization of replication domains during embryonic stem cell differentiation. *PLoS Biology* *, 6* (10), 2220-36.

Hough, P. V. (1962). *Patent No. 3,069,654.* U.S.A.

Illingworth, J., & Kittler, J. (1988). A survey of the Hough transform. *Computer Vision, Graphics, and Image Processing* *, 44* (1), 87-116.

Lipson, D., Aumann, Y., Ben-Dor, A., Linial, N., & Yakhini, Z. (2006). Efficient calculation of interval scores for DNA copy number data analysis. *Journal of Computational Biology* *, 13* (2), 215-228.

Olshen, A. B., Venkatraman, E. S., Lucito, R., & Wigler, M. (2004). Circular binary segmentation for the analysis of array-based DNA copy number data. *Biostatistics* *, 5* (4), 557-572.

Thrift, P. R., & Dunn, S. M. (1983). Approximating point-set images by line segments using a variation of the Hough Transform. *Computer Vision, Graphics and Image Processing* *, 21* (3), 383-394.
