# Supplementary figures and images for: Systematic Determination of Replication Activity Type Highlights Interconnections between Replication, Chromatin Structure and Nuclear Localization
Source: PLoS One. 2012 Nov 7;7(11):e48986. doi: 10.1371/journal.pone.0048986 (PMC3492150; doi:10.1371/journal.pone.0048986)

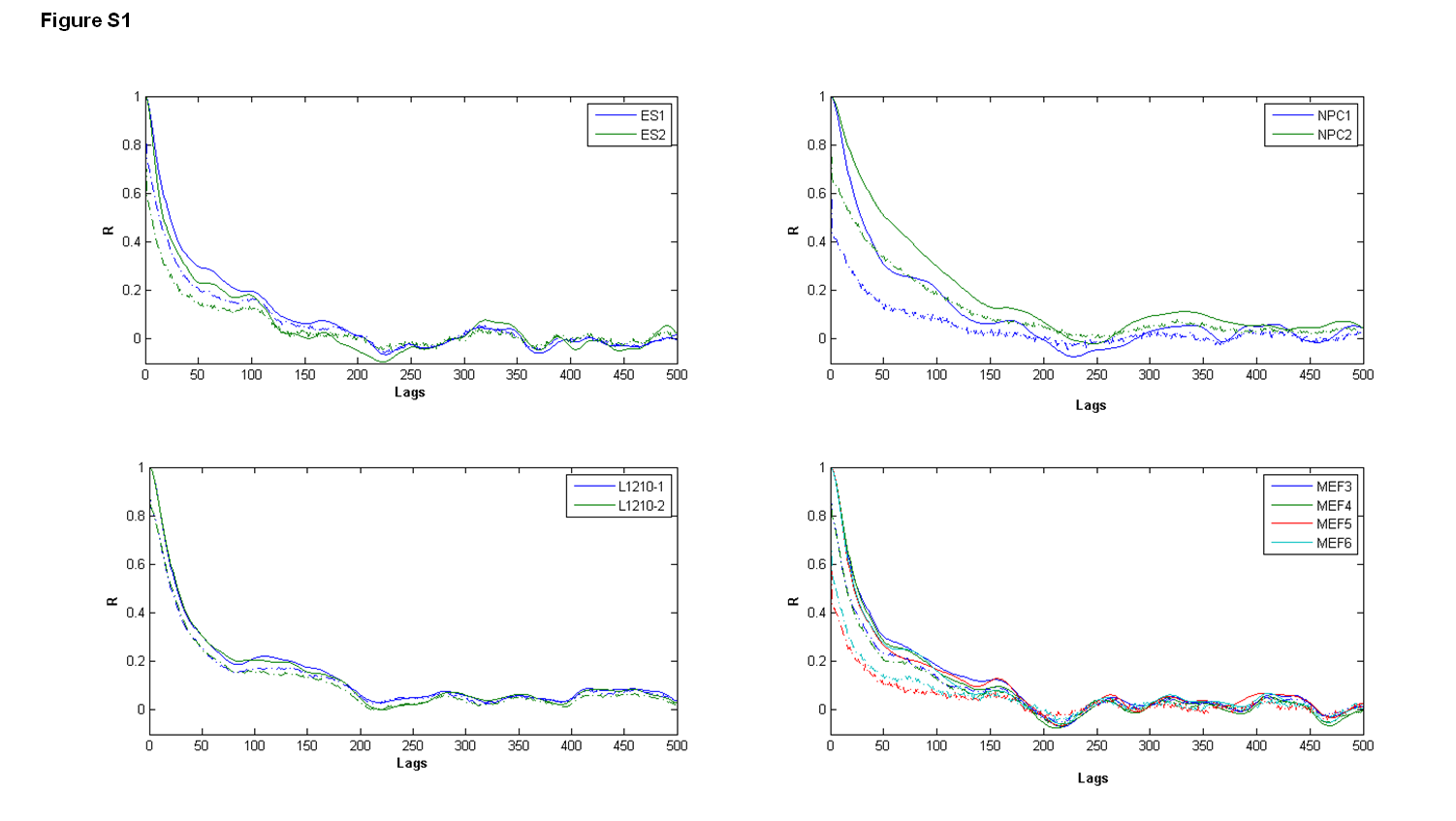

Supplement: Figure S1 — ToR Autocorrelation. Autocorrelation was calculated for all probes on chromosome 1 (after sorting) before and after segmentation (dashed and solid lines respectively). The probe spacing was on average 40 kb. A significant autocorrelation is observed for all tissues at least until lag = 100. Note that the autocorrelation is the smallest for the ES cells (and also NPC1 which resembles ES cells, see also S3) which suggests smaller replication zones in these cells. (TIF) [file pone.0048986.s002.tif]

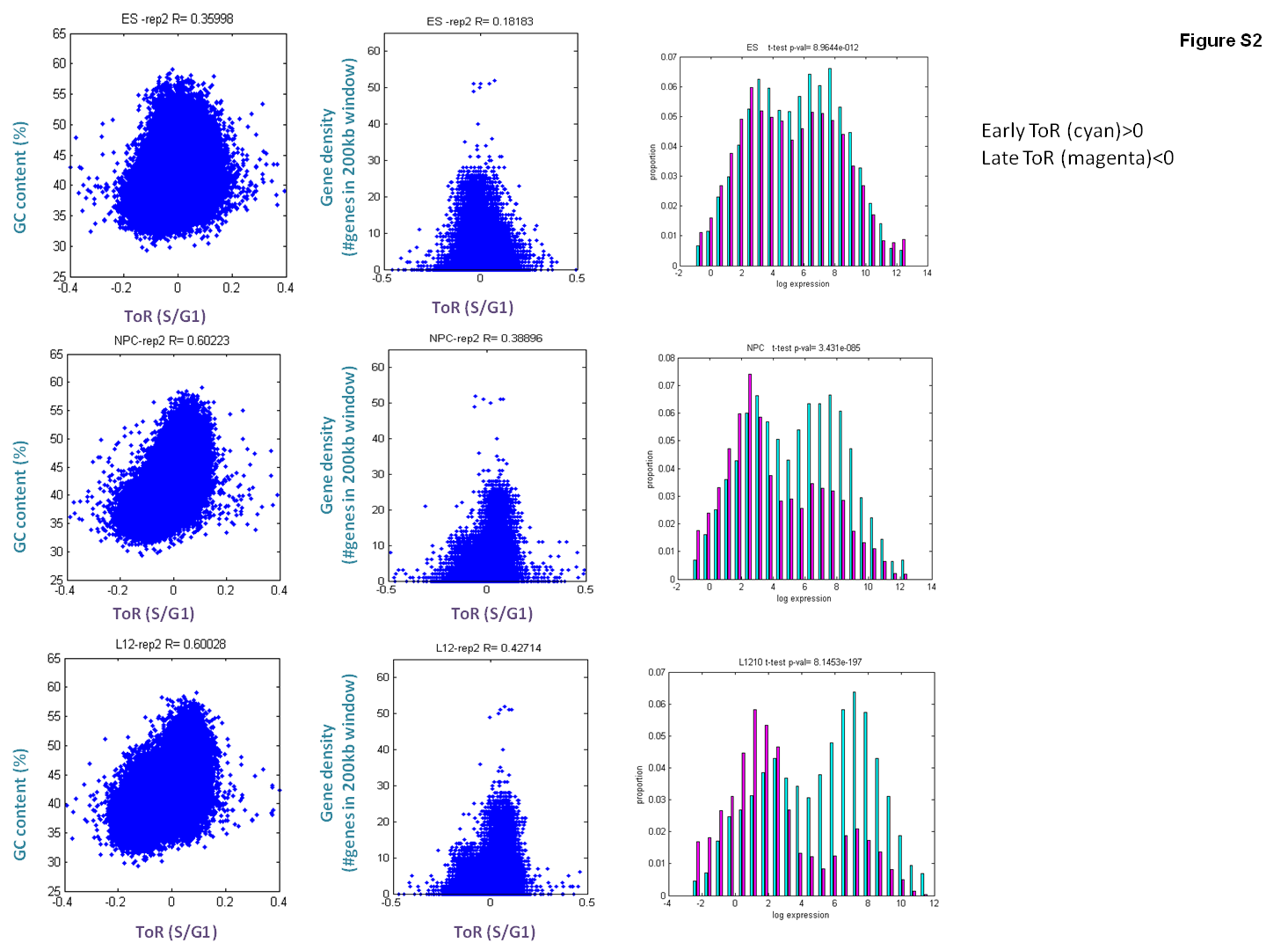

Supplement: Figure S2 — Correlation between ToR, genomic features and expression. Representative plots of the ToR against GC content (left panels) and gene density (middle panel) are shown. The cell type (ES, upper row, NPC middle rows and L1210, bottom rows) and the Pearson correlation coefficient are written above each graph. The right plots shows the difference in the expression distribution between early (cyan) and late (blue) genes. The statistical significance of the difference between the distributions (Student T test) are written above each plot. Note that the correlation between ToR and GC content/gene density is high and significant in all cell types, however, in stem cells the correlation is the lowest. (TIF) [file pone.0048986.s003.tif]

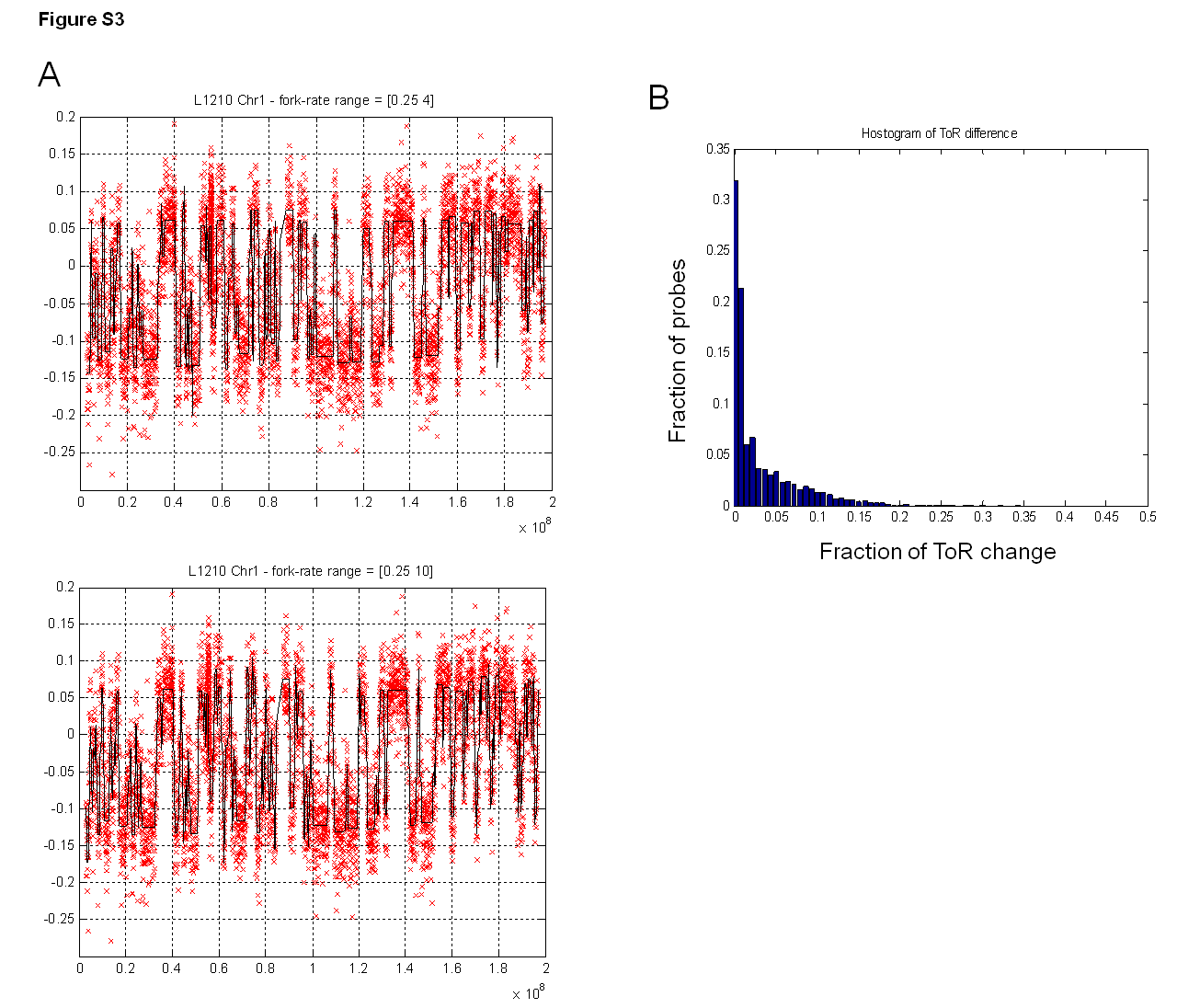

Supplement: Figure S3 — The effect of relaxing fork rate assumption on ARTO results. We tested ARTO using two different fork rate ranges, namely 0.25–4 Kb/minute (A top panel), and 0.25–10 Kb/minute (A bottom panel). B shows the distribution of differences in the results. Note that for >80% of the probes inferred ToR was affected by less than 5%. Our test also shows that 4.5% of probes changed from CTR to TTR and no probe changed from TTR to CTR. (TIF) [file pone.0048986.s004.tif]

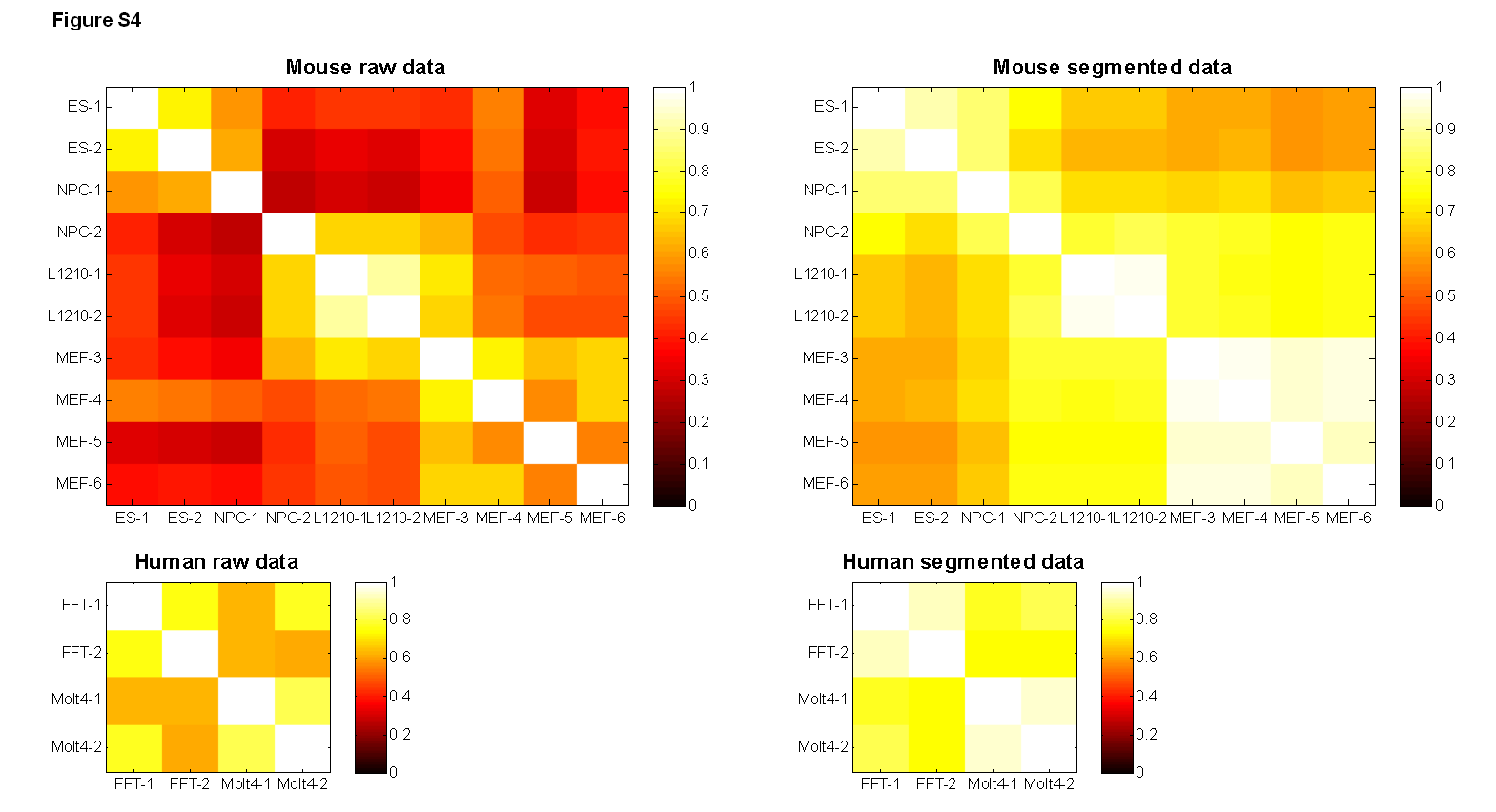

Supplement: Figure S4 — ToR Correlation before and after segmentation. Up-left: correlation of raw ToR measurements between mouse tissues. Up-right: correlation of segmented ToR between mouse tissues. Down-left: correlation of raw ToR measurements between human tissues. Down-right: correlation of segmented ToR between human tissues. As expected, the correlations between replicates of the same tissue are higher than the correlation between tissues (except in mouse NPC). The correlation is greatly improved after segmentation. (TIF) [file pone.0048986.s005.tif]

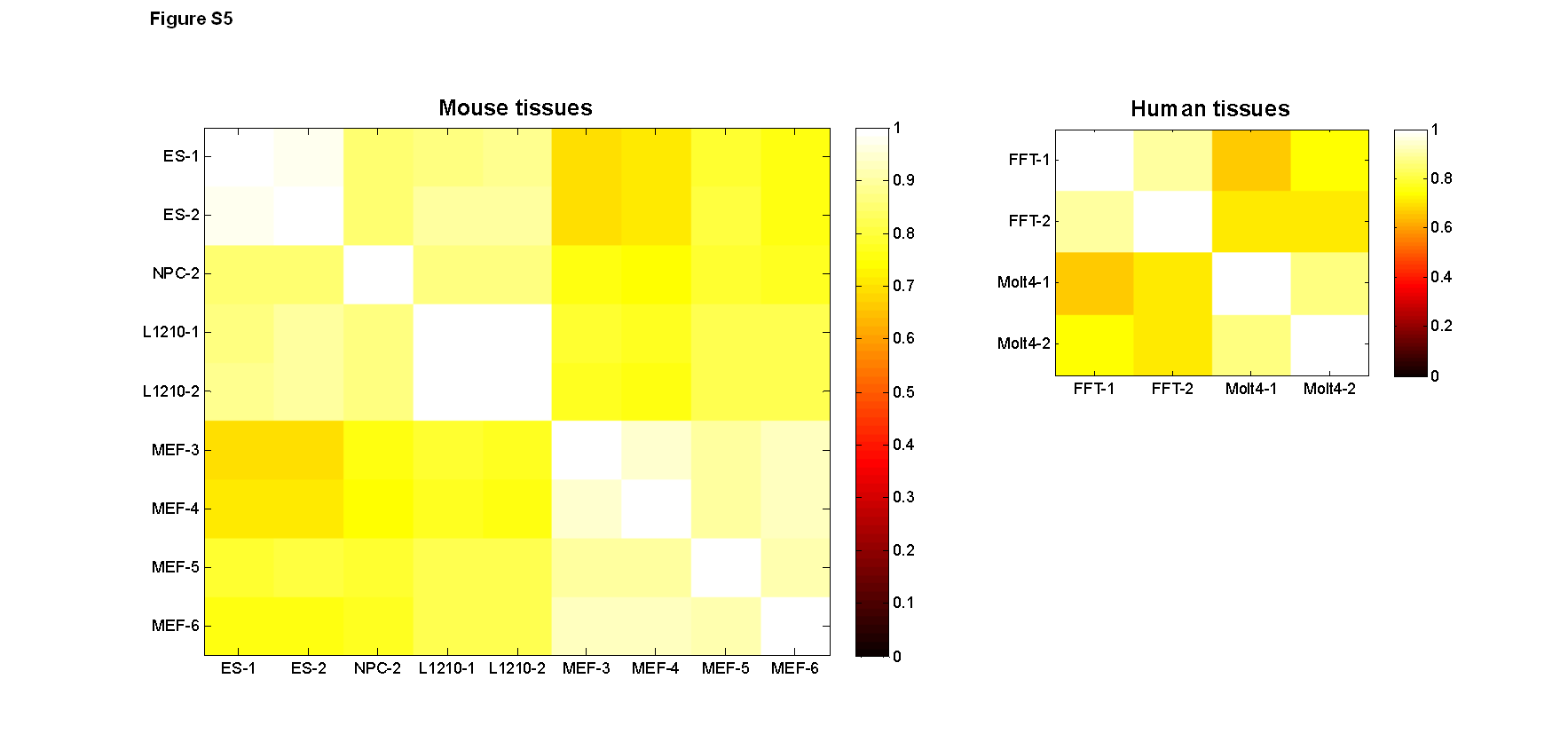

Supplement: Figure S5 — Comparison of replication activity type between tissues. Percentage of probes with identical replication activity type assignment (CTR or TTR) for each pair of mouse (on the left) and human (on the right) tissues. Replicates of the same tissue show a higher percentage of identical assignments. (TIF) [file pone.0048986.s006.tif]

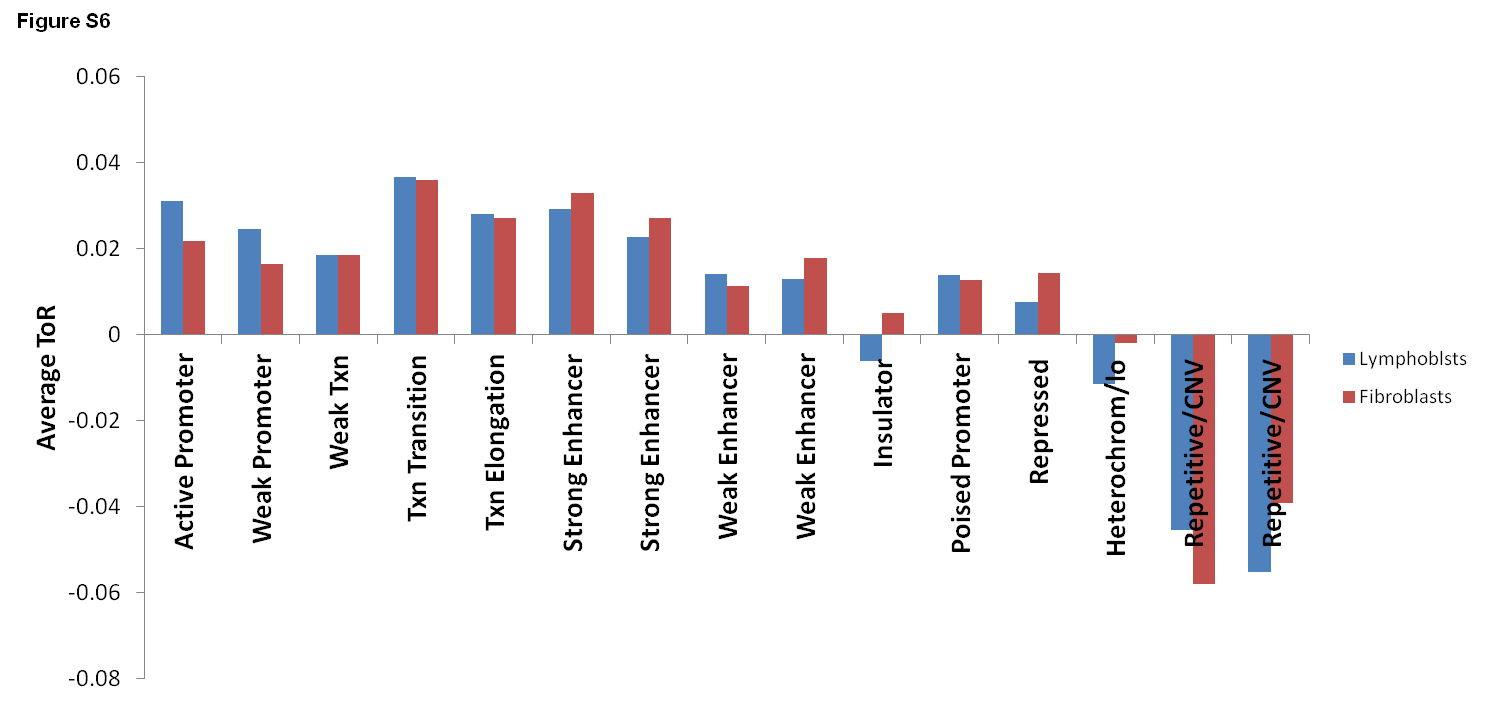

Supplement: Figure S6 — ToR distribution in 15 chromatin states. The average ToR (S/G1 log ratio) in the regions associated with each of the 15 distinct chromatin states in lymphoblasts (blue) and fibriblasts (red) published by Ernst et al. (Nature 473, 43–49, 2011). Early replication is enriched for promoter and transcription states, whereas late replication is enriched for genomic regions characterized by the repressive and repetitive states. (TIF) [file pone.0048986.s007.tif]

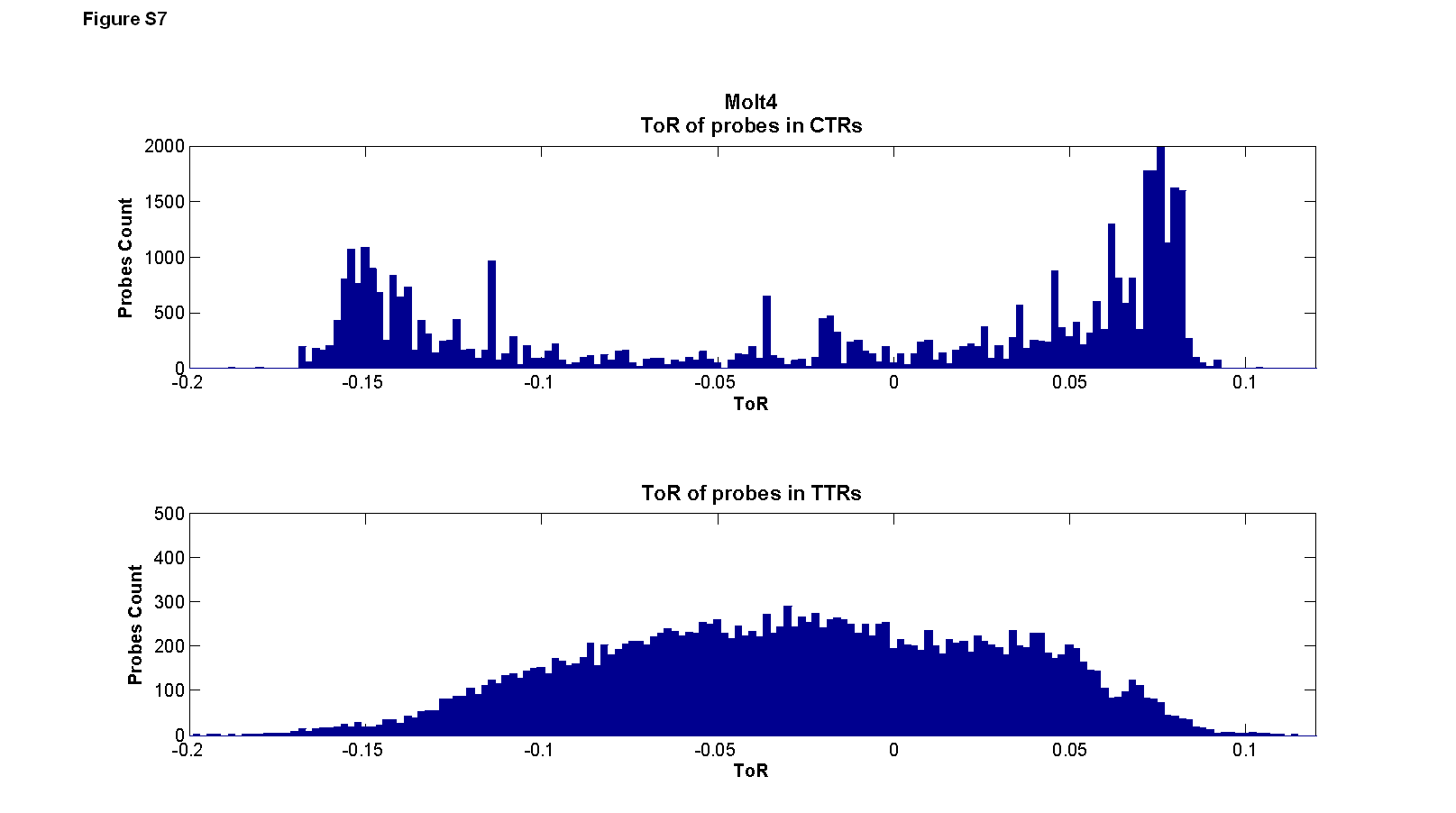

Supplement: Figure S7 — comparison of ToR distribution between CTRs and TTRs. ToR distribution of all the probes in CTRs (upper panel) and TTRs (lower panel), in human lymphoblasts. The ToR distribution is very different between the two types of regions – most CTRs replicate early or late, while TTR ToR is mostly in middle S-phase. (TIF) [file pone.0048986.s008.tif]

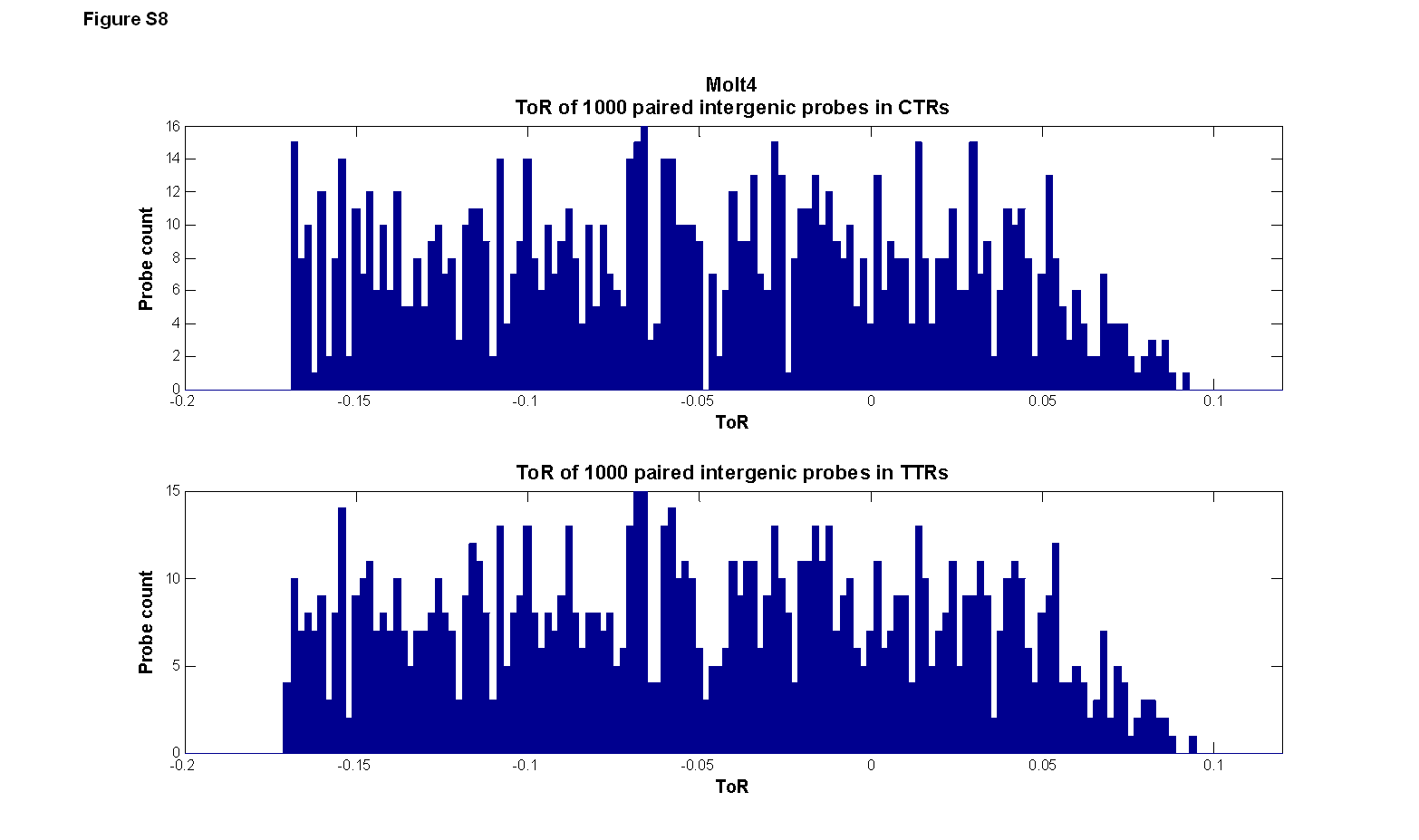

Supplement: Figure S8 — comparison of ToR distribution in the paired probes of CTRs and TTRs. ToR of 1000 paired intergenic probes in CTRs (upper panel) and TTRs (lower panel), in human lymphoblasts. The probes were randomly selected so that the ToR distribution of both sets of probes will be the same and close to uniform. (TIF) [file pone.0048986.s009.tif]

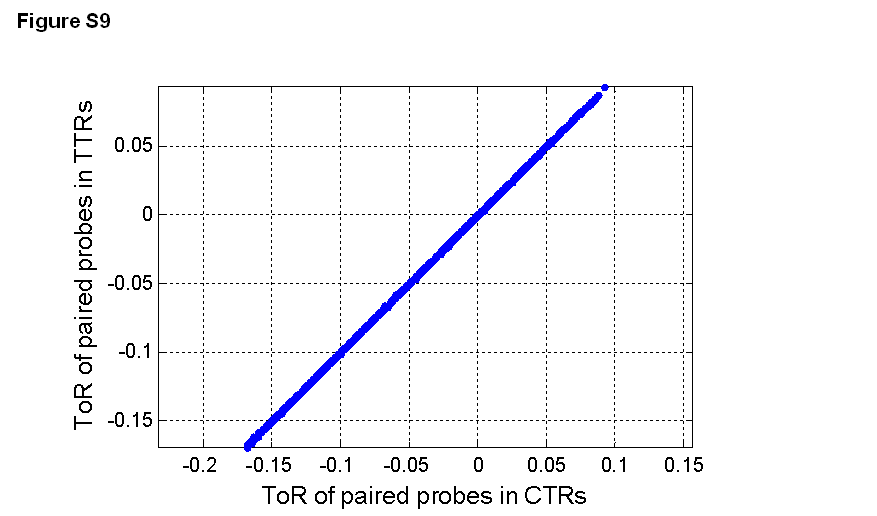

Supplement: Figure S9 — ToR of paired probes. Scatter plot of the ToR in 1000 paired intergenic probes in CTRs (x-axis) and TTRs (y-axis), in human lymphoblasts. Each pair of matched probes has the same ToR. (TIF) [file pone.0048986.s010.tif]

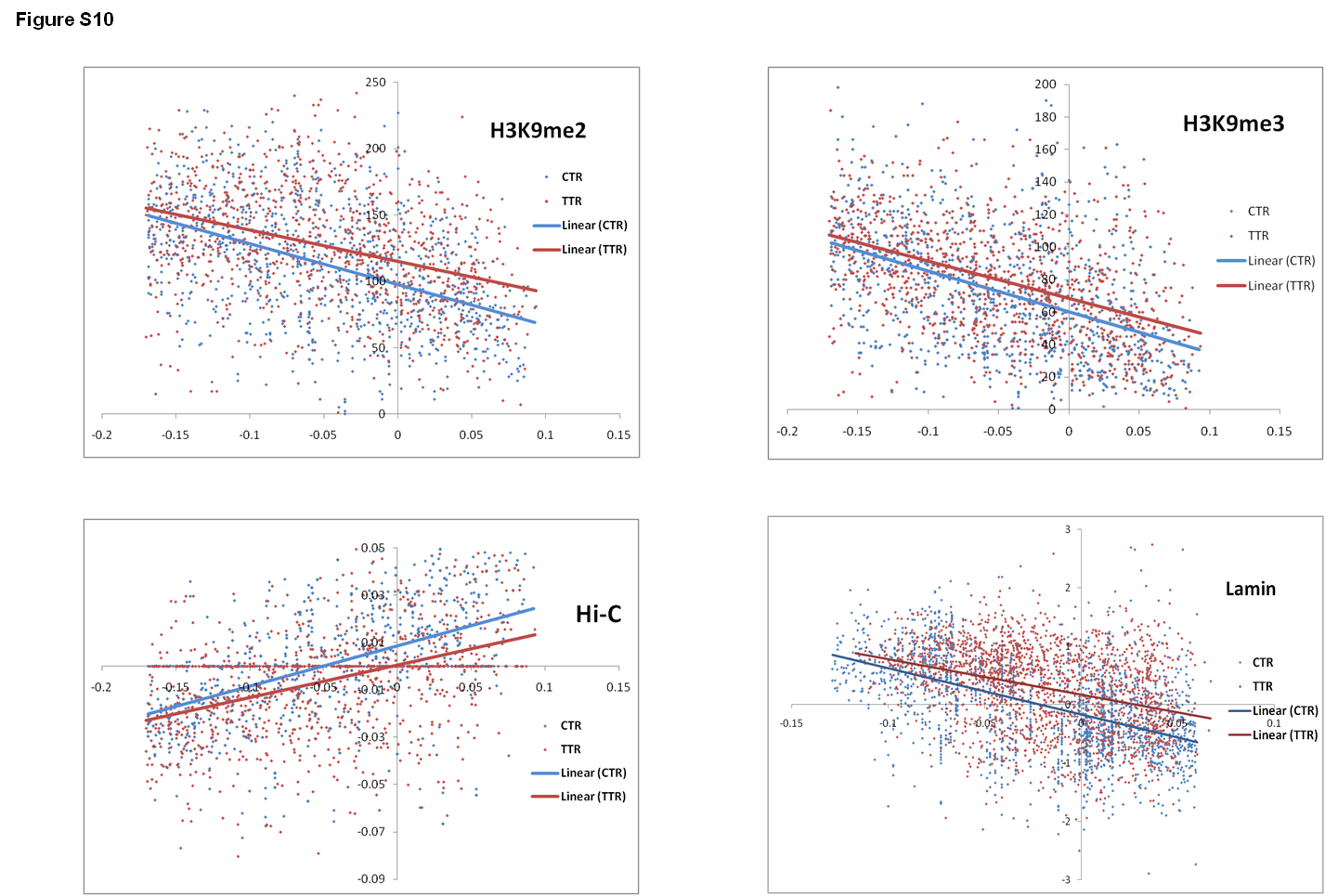

Supplement: Figure S10 — CTRs and TTRs correlation with chromatin structure. Scatter plots representing the correlations between the ToR and various chromatin features are shown for 1000 probes residing in CTRs (blue) and TTRs (red) regions with matched ToR. In all plots, x-axis represents ToR. Note that the ToR is positively correlated for the HiC data (In which higher values are for open chromatin) and negatively correlated with the other repressive markers both in TTRs and CTRs. However the CTRs values are consistently lower for repressive markers and higher for activation markers suggesting that TTRs are packed in closer chromatin than CTRs. (TIF) [file pone.0048986.s011.tif]

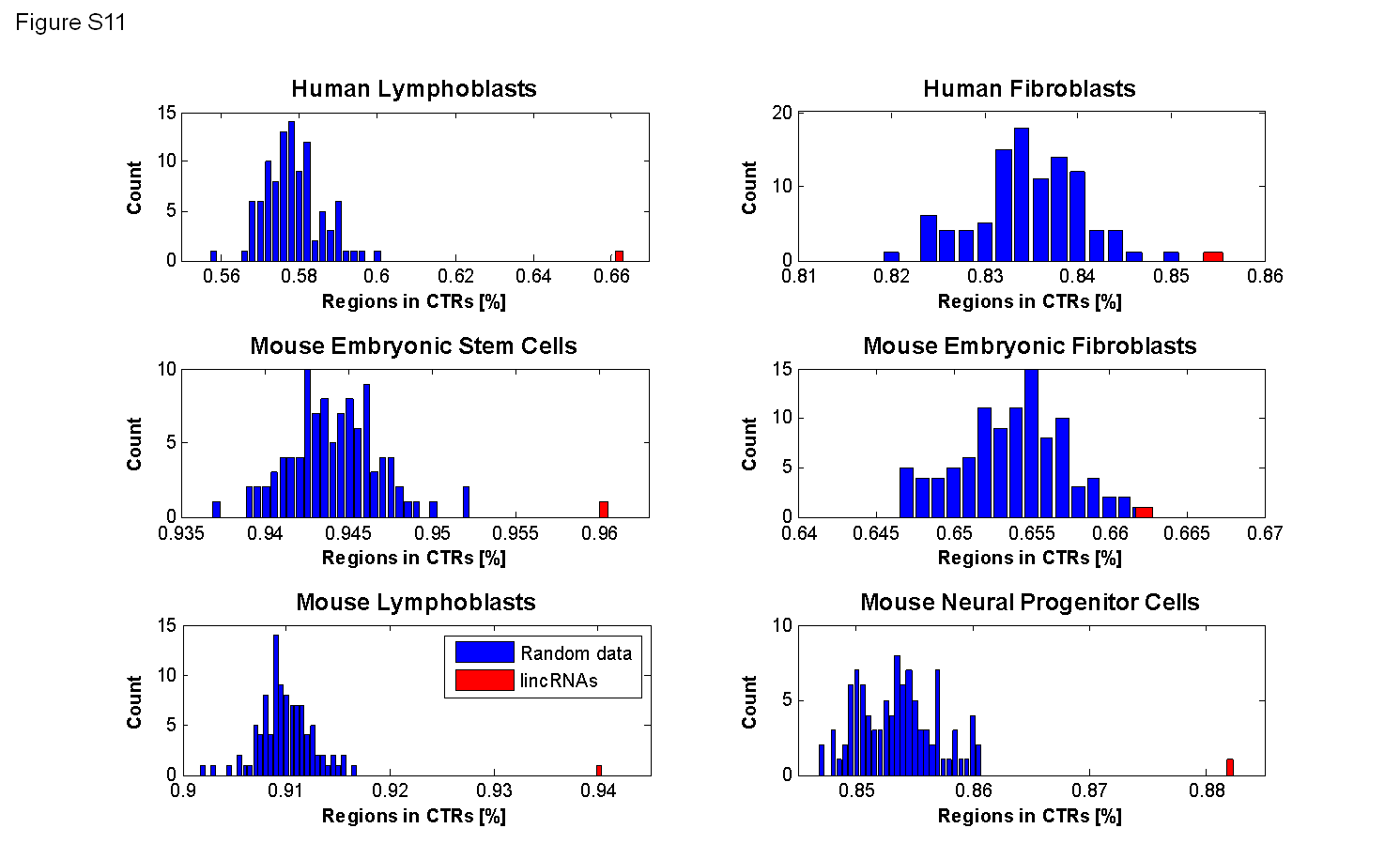

Supplement: Figure S11 — LincRNA transcribed exons have a tendency to reside in CTRs. For each tissue type, the percentage of lincRNA transcribed exons that reside in CTRs is marked in red. Histograms of the percentage of randomly drawn genomic regions (with similar properties as lincRNA exons) assigned to CTRs in 100 random sets are marked with blue. In all mouse and human tissues, lincRNA transcribed exons have a tendency to reside in CTRs, more than would be expected from the random control. (TIF) [file pone.0048986.s012.tif]

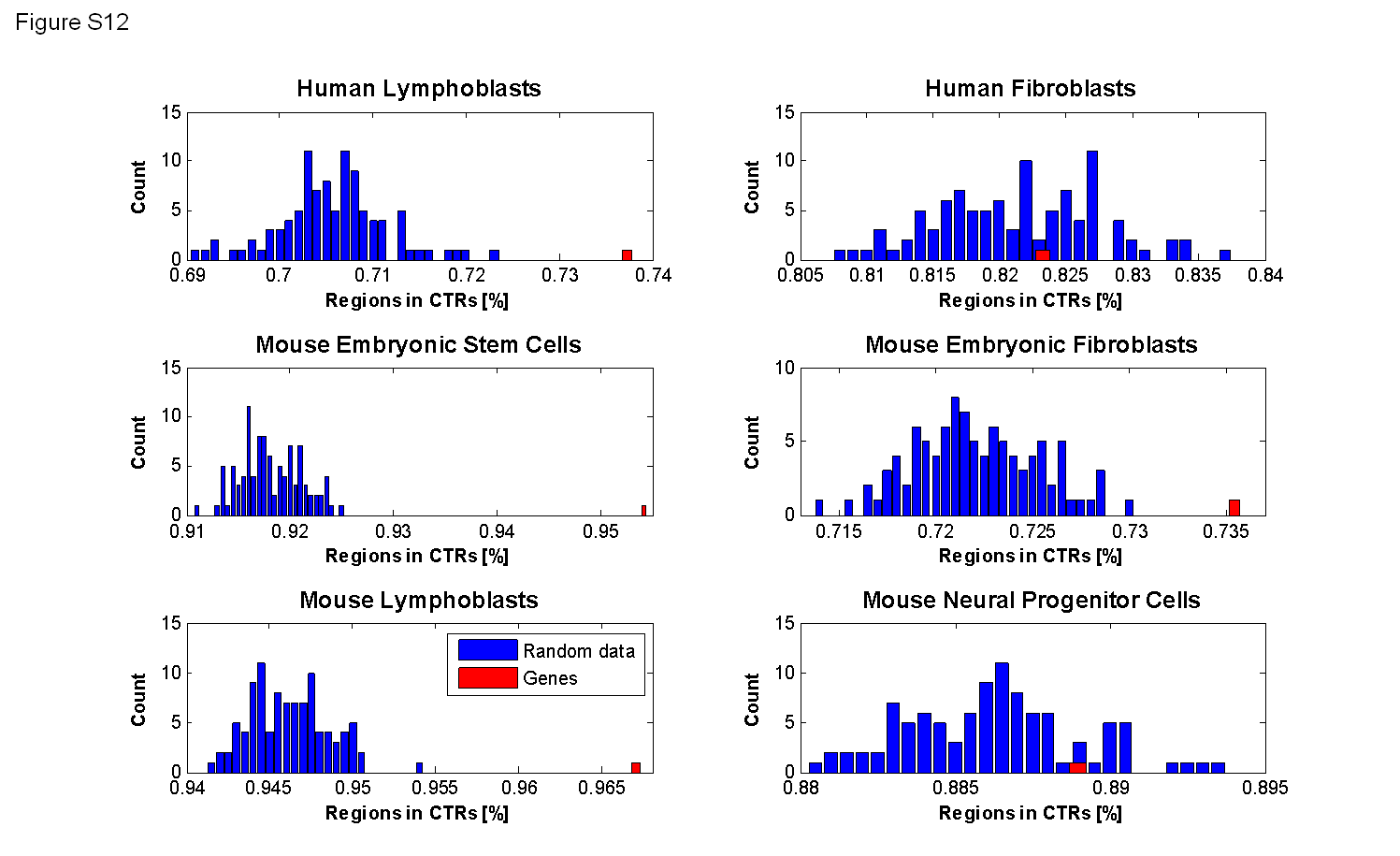

Supplement: Figure S12 — Expressed genes have a tendency to reside in CTRs. For each tissue type, the percentage of expressed genes that reside in CTRs is marked in red. Histograms of the percentage of randomly drawn genomic regions (with similar properties as lithe genes) assigned to CTRs in 100 random sets are marked with blue. In most tissues (except for human fibroblasls and mouse NPC) expressed genes have a tendency to reside in CTRs, more than would be expected from the random control. (TIF) [file pone.0048986.s013.tif]

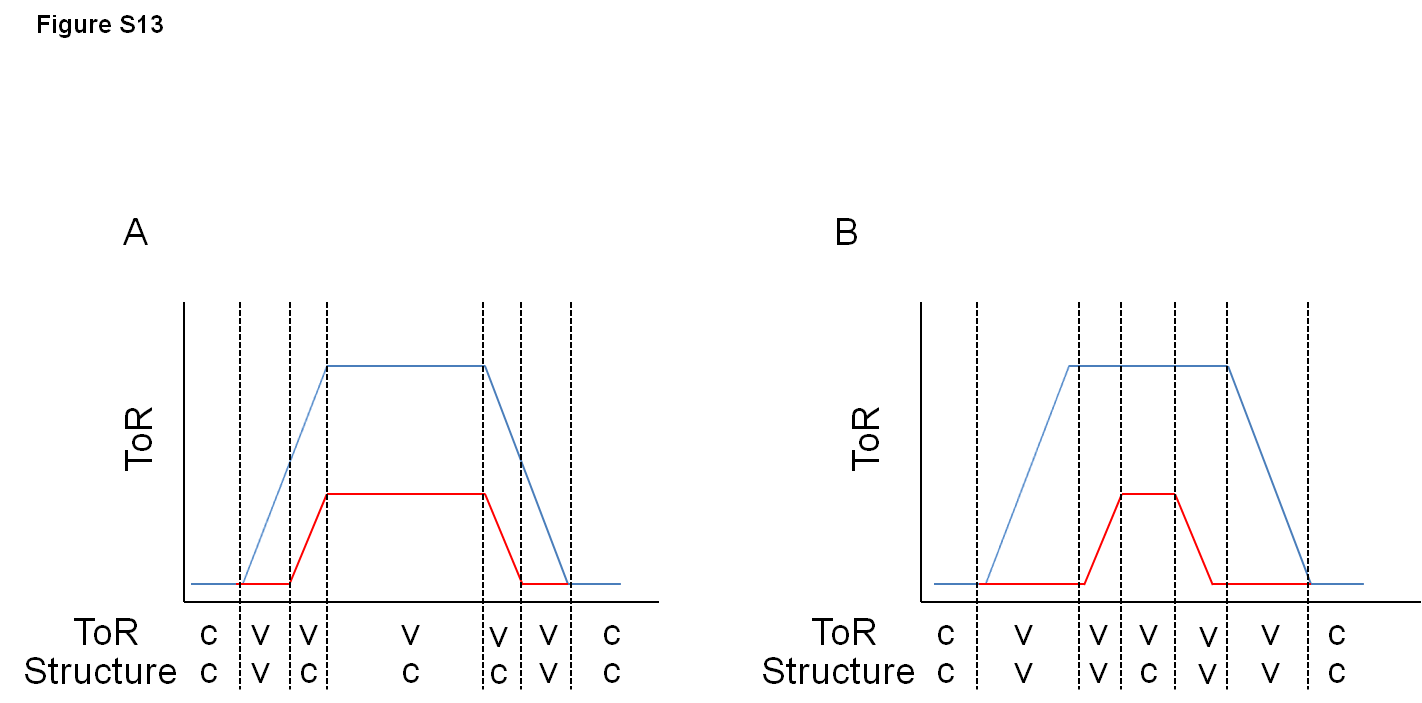

Supplement: Figure S13 — Associations between changes in the ToR and in the replication activity type. Schematic representations of ToR maps in two regions in which the ToR is different between two tissues (red and blue). The two regions differ in the extent of ToR change (in A the change of the ToR affects an entire CTR whereas in B it was limited to a small portion of the CTR). The dotted lines separate sub regions according to their replication activity type. The letters below the graphs indicate for each segment whether its ToR and its structure was constant (c) or variable (v) between the two tissues. Note that in B most of the regions that changed their ToR also changed their structure whereas in A ToR change with constant structure is much more common. (TIF) [file pone.0048986.s014.tif]

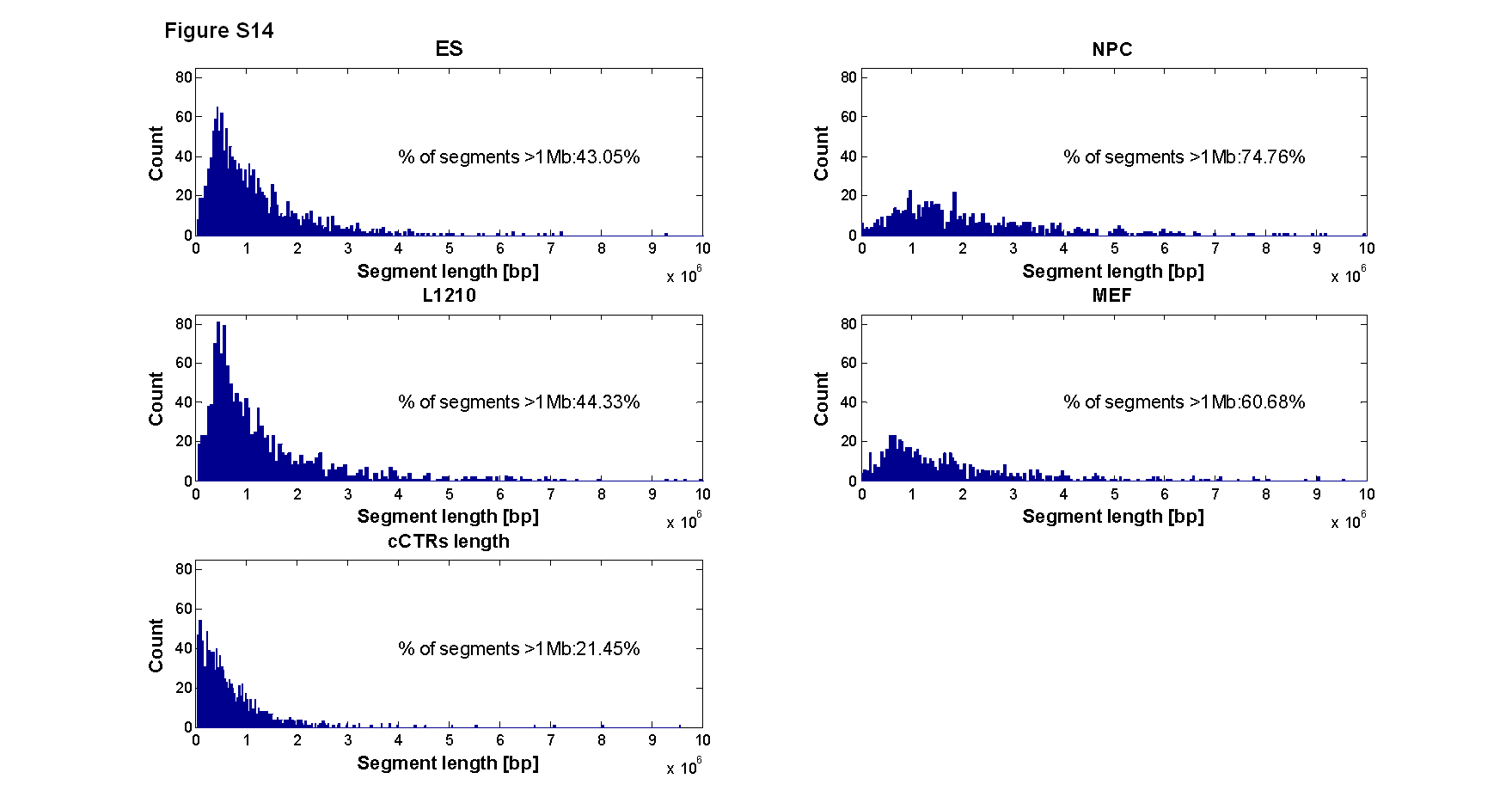

Supplement: Figure S14 — Distribution of CTR segments lengths in mouse tissues. The distribution of CTR segments lengths (in bp) for all four mouse tissue types, as well as of cCTRs (constant CTRs, which are regions that are defined as CTRs in all four mouse tissues). In each histogram, the percentage of regions longer than 1 Mb is written. (TIF) [file pone.0048986.s015.tif]

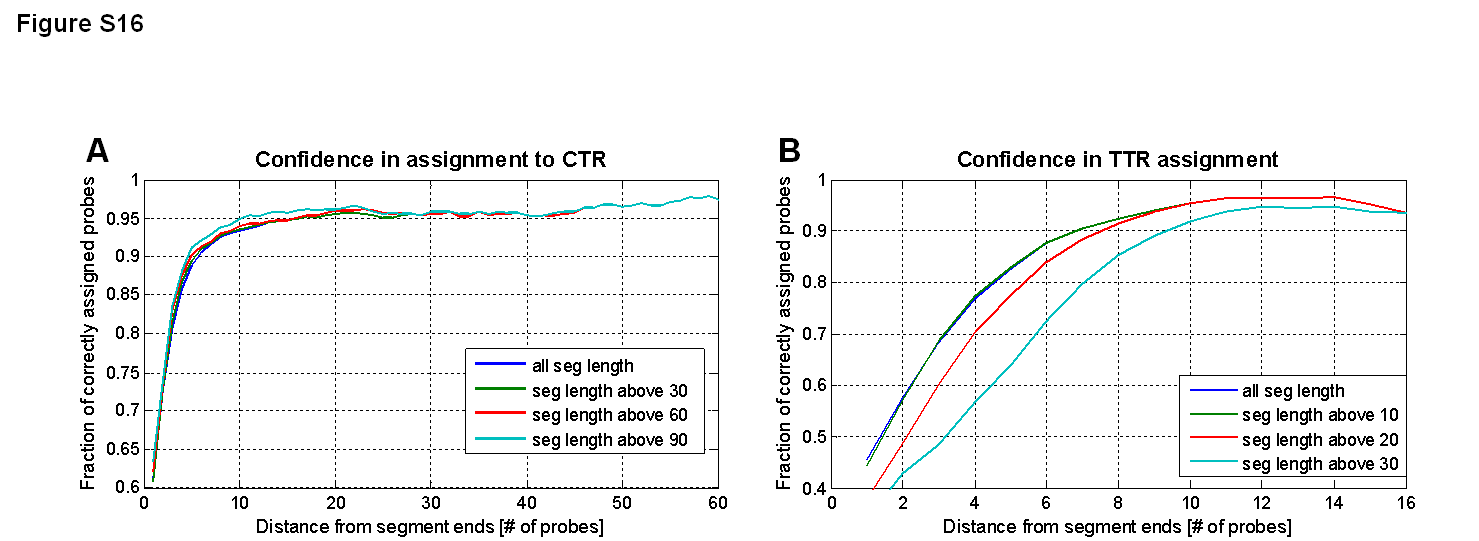

Supplement: Figure S16 — Confidence level in CTR and TTR assignments vs. distance from segment ends. The fraction of correctly assigned probes as a function of distance from segment ends (measured in probes) in simulated data, reflecting the confidence level of replication activity type assignment for both CTR (A) and TTR (B). The averaging is done on different segments length, for example for CTRs: all segments and segments with lengths over 30, 60 and 90 probes. (TIF) [file pone.0048986.s017.tif]
